# Augmented weighted K-means grey wolf optimizer: An enhanced metaheuristic algorithm for data clustering problems

**DOI:** 10.1038/s41598-024-55619-z

**Published:** 2024-03-05

**Authors:** Manoharan Premkumar, Garima Sinha, Manjula Devi Ramasamy, Santhoshini Sahu, Chithirala Bala Subramanyam, Ravichandran Sowmya, Laith Abualigah, Bizuwork Derebew

**Affiliations:** 1 Department of Electrical & Electronics Engineering, Dayananda Sagar College of Engineering, Kumaraswamy Layout, Bengaluru, Karnataka 560078 India; 2 Department of Computer Science and Engineering, Jain University, Ramanagaram, Bengaluru, Karnataka India; 3 Department of Computer Science and Engineering, KPR Institute of Engineering and Technology, Coimbatore, Tamil Nadu India; 4 Department of Computer Science & Engineering, GMR Institute of Technology, Rajam, Srikakulam, Andhra Pradesh India; 5 Department of Computer Science and Engineering, Vardhaman College of Engineering, Hyderabad, India; 6 Department of Electrical and Electronics Engineering, Manipal Institute of Technology, Manipal Academy of Higher Education, Manipal, Karnataka India; 7 Computer Science Department, Al al-Bayt University, Mafraq, 25113 Jordan; 8 Artificial Intelligence and Sensing Technologies (AIST) Research Center, University of Tabuk, 71491 Tabuk, Saudi Arabia; 9 Hourani Center for Applied Scientific Research, Al-Ahliyya Amman University, Amman, 19328 Jordan; 10 MEU Research Unit, Middle East University, Amman, 11831 Jordan; 11 Department of Electrical and Computer Engineering, Lebanese American University, Byblos, 13-5053 Lebanon; 12 School of Engineering and Technology, Sunway University Malaysia, 27500 Petaling Jaya, Malaysia; 13 College of Engineering, Yuan Ze University, Taoyuan, Taiwan; 14 Applied science research center, Applied science private university, Amman, 11931, Jordan; 15 Department of Statistics, College of Natural and Computational Science, Mizan-Tepi University, Tepi Bushira, Ethiopia

**Keywords:** Computational intelligence, Data mining, Grey wolf optimizer, K-means clustering, Optimization algorithm, Engineering, Mathematics and computing

## Abstract

This study presents the K-means clustering-based grey wolf optimizer, a new algorithm intended to improve the optimization capabilities of the conventional grey wolf optimizer in order to address the problem of data clustering. The process that groups similar items within a dataset into non-overlapping groups. Grey wolf hunting behaviour served as the model for grey wolf optimizer, however, it frequently lacks the exploration and exploitation capabilities that are essential for efficient data clustering. This work mainly focuses on enhancing the grey wolf optimizer using a new weight factor and the K-means algorithm concepts in order to increase variety and avoid premature convergence. Using a partitional clustering-inspired fitness function, the K-means clustering-based grey wolf optimizer was extensively evaluated on ten numerical functions and multiple real-world datasets with varying levels of complexity and dimensionality. The methodology is based on incorporating the K-means algorithm concept for the purpose of refining initial solutions and adding a weight factor to increase the diversity of solutions during the optimization phase. The results show that the K-means clustering-based grey wolf optimizer performs much better than the standard grey wolf optimizer in discovering optimal clustering solutions, indicating a higher capacity for effective exploration and exploitation of the solution space. The study found that the K-means clustering-based grey wolf optimizer was able to produce high-quality cluster centres in fewer iterations, demonstrating its efficacy and efficiency on various datasets. Finally, the study demonstrates the robustness and dependability of the K-means clustering-based grey wolf optimizer in resolving data clustering issues, which represents a significant advancement over conventional techniques. In addition to addressing the shortcomings of the initial algorithm, the incorporation of K-means and the innovative weight factor into the grey wolf optimizer establishes a new standard for further study in metaheuristic clustering algorithms. The performance of the K-means clustering-based grey wolf optimizer is around 34% better than the original grey wolf optimizer algorithm for both numerical test problems and data clustering problems.

## Introduction

### Basic concepts of data clustering

An unsupervised learning technique called clustering separates a database into clusters of identical matters by reducing the resemblance among objects in diverse groups and exploiting the similarities between entities in the identical cluster^1–3^. Clustering has been a crucial tool for data analysis in many disciplines, including intrusion detection, data mining, bioinformatics, and machine learning systems. Clustering is also used in various fields, including social network analysis, robotics, and networks. Hierarchical clustering, mixed clustering, learning network clustering, and partition clustering can all be used to group data into clusters^4–6^. The main objective of clustering techniques is to make clusters more homogeneous and heterogeneous. Partitional clustering techniques previously ran into issues such as responsiveness to the starting center points, local optima trap, and lengthy run times. Clustering separates a set of datasets with $d$-dimensions into $k$ different groups. Every division is known as a cluster ${C}_{i}$. Each cluster's members share several traits in common, although there is little overlap between them^7–9^. In this situation, clustering's objective is to identify the separate groups and allocate objects depending on how closely they resemble the appropriate groups. The absence of initial tags for observations is the primary distinction between the clustering and classification methods. However, classification techniques use predetermined classifications to which objects are given, clustering groups of objects without previous knowledge^10–13^.

Numerous research efforts on data clustering have been offered throughout the past decades. To cluster a dataset, there are various solutions to the clustering problem. These methods primarily use complicated network approaches, $K$-means, and its improved variants, metaheuristic algorithms, and other methods^14–18^. One of the most well-known of these methods is the $K$-means algorithm, which attempts to partition a complete dataset into $k$ clusters by randomly selecting $k$ data points as starting cluster centers. The $K$-means method, however, is delicate to the choice of beginning points and might be unable to group huge databases. Many experts have concentrated on swarm intelligence algorithms to address the drawbacks of the K-means technique, which can do a simultaneous search in a complicated search space to avoid a premature convergence trap^3,15,19–24^. Researchers also focused on merging metaheuristic techniques with conventional clustering techniques to lessen such limitations.

### Literature review

Metaheuristics algorithms are population-based and imitate the shrewd behaviour of socially organized creatures. Glowworm and crow search-based clustering have been proposed for data clustering. These techniques depicted the clustering solutions as swarms of creatures. Then, to quickly cover the search space, these strategies use clever intensification and diversification techniques. Although metaheuristic algorithms reduced classic clustering techniques' execution times, they still had drawbacks^25^. The shortcomings of Particle Swarm Optimization (PSO) and its competitors can be summarized as follows: lack of developed memory elements and the diversity of populations^26^. The PSO and its variants use a single optimal solution stored in the solution space to reposition the members of the swarm, which can cause them to become stuck in local minima. These shortcomings caused PSO and its variants to obtain solutions with low quality and convergence speed, which accounts for the birth of numerous other algorithms reported in the literature^13,27,28^.

The authors of Ref.^29^ suggested a genetic algorithm (GA) for clustering that exploits a region-based crossover mechanism. It finds the best preliminary center for the $k$-means algorithm. The chromosomes translate the clusters of centroids, and during the crossover, chromosome pairs exchange several centroids located in the same area of space. According to experimental research, the region-based crossover outperforms a random exchange of centroids. The authors of Ref.^2^ suggested a differential evolution (DE) algorithm integrating the $k$-means technique. The local search and initial population are conducted using the $k$-means algorithm. The population vectors set the cluster centroids. In an additional effort to eliminate the redundant nature of the centroid encoding, The authors of Ref.^30^ reported a technique that hybridized the $k$-means algorithm and gravitational search algorithm. The $k$-means was used to improve the generation of the initial population; one individual was generated using $k$-means, and the remaining individuals were generated randomly. A data clustering approach based on the Gauss chaotic map-using PSO was presented in Ref.^31^. Sequences produced by the Gauss chaotic drift were used to replace the random elements affecting the velocity update's cognitive and social components. A cooperative artificial bee colony (CABC) approach for data clustering was proposed in Ref.^32^, in which every bee contributes to creating the optimal solution. The appropriate solution for each bee is thought to replace every solution of the optimal solution. The authors of Ref.^33,34^ used representative points, typically not centroids, to indicate potential solutions, and, as with centroids, a dataset partition was created by allocating data to the cluster closest to the representative point.

Many metaheuristic algorithms have recently been reported in addition to the above-discussed algorithms for numerical and real-world engineering design optimization problems, including data clustering. For instance, ant colony optimization^35^, firefly algorithm^36,37^, flower pollination algorithm^38^, grey wolf optimizer (GWO)^39–42^, Jaya algorithm^43^, Teaching–learning based optimization (TLBO) algorithm^44^, Rao algorithm^45^, political optimizer ^46^, whale optimization algorithm (WOA)^47^, Moth flame algorithm (MFO)^48^, multi-verse optimizer (MVO)^49^, Salp swarm algorithm (SSA)^50,51^, spotted hyena optimizer^52^, butterfly optimization^53^, lion optimization^54^, fireworks algorithm^55^, Cuckoo search algorithm^56^, bat algorithm^57^, Tabu search^58^, harmony search algorithm^59^, Newton–Raphson optimizer^60^, reptile search algorithm^61^, slime mould algorithm^62,63^, harris hawk optimizer^64^, Chimp optimizer^65^, artificial gorilla troop optimizer^66^, atom search algorithm^67^, marine predator algorithm^68,69^, sand cat swarm algorithm^70^, equilibrium optimizer^71,72^, Henry gas solubility algorithm (HGSA)^73^, resistance–capacitance algorithm^74^, arithmetic optimization algorithm^75^, quantum-based avian navigation optimizer^76^, multi trail vector DE algorithm^10,77^, arithmetic optimization algorithm^78^, starling murmuration optimizer^79^, atomic orbit search (AOS)^80^, subtraction-average-based optimizer^81^, etc. are reported for solving optimization problems. In conclusion, these new algorithms and their improved variations based on different metaheuristic computing algorithms yield greater results than before^82–84^. A comparative study to show the recent efforts in using metaheuristic algorithms for data clustering is listed in Table 1.

**Table 1:** Summary of a few metaheuristic algorithms applied to data clustering.

Algorithm	Year	Inspiration	Remarks
GA with gene rearrangement^29^	2009	GA with gene rearrangement is reported, i.e. a new crossover operator is introduced to improve the exploitation	This algorithm has been tested only for image clustering
CABC^32^	2010	Inspired by the foraging behaviour of honey bees	Six real-time datasets are used to test he algorithm. The comparison was made between GA and PSO
Gravitational search algorithm^30^	2011	Inspired by the gravity law and mass interactions	The comparison was made between PSO and GA only. Detailed analysis is not available
DE^2^	2013	Inspired by Darwin's theory of evolution	The comparison is carried out between different variants of DE
PSO hybridized with magnetic charge system search^27^	2015	Hybrid PSO with magnetic charge system searches and is inspired by electromagnetic theory	Validation is carried out for very few benchmark datasets
Glowworm optimization algorithm^25^	2017	The swarm's movement of glowworms is determined by their distance from one another and by a luminous quantity	Detailed analysis is not carried out for the clustering problems
Symbiotic organism search algorithm^85^	2019	Inspired by the symbiotic interaction implemented to survive and propagate	Ten datasets are used to validate the algorithm and compared with PSO and GA
GWO^39^	2020	Inspired by the social hierarchy and hunting behaviour of the grey wolves	Limited datasets are used for validation
MFO^86^	2021	Inspired by the moth's intelligence, i.e., transverse orientation to navigate in nature	Original MFO is applied, and it gets trapped by local optima
Aquila optimizer^87^	2022	Hybridized with the arithmetic optimization algorithm. The Aquila optimizer has inspired the behaviours during the finding of the prey	It has been applied for text and data clustering problems. But computation complexity is high
Chaos-based PSO^31^	2022	Inspired by PSO and Gaussian Chao map	It has been applied for auto labelling in recognition of human activity

The authors of Ref.^88^ proposed an improved version of the firefly algorithm by hybridizing the exploration method and a chaotic local search approach. The improved firefly algorithm was practically validated for routinely choosing the optimal dropout rate for the regularization of the neural networks. In order to maximize the local and global characteristics collected from each of the handwritten phrase representations under consideration, a hierarchical feature selection framework based on a genetic algorithm has been developed in Ref.^89^. The authors of Ref.^90^ have reviewed the PSO algorithm and its variants for medical disease detection. The overfitting problem was addressed in Ref.^91^ by using the sine-cosime algorithm to determine an appropriate value for the regularisation parameter dropout. According to the literature review, swarm strategies are currently being utilized effectively in this domain, although their future application has not yet been fully explored. The effective extreme gradient boosting classification algorithm, which is used to classify the characteristics obtained by the convolutional layers, was used to substitute the fully connected layers of a standard convolution neural network in order to increase classification accuracy^92^. Furthermore, to support the suggested research, a hybrid version of the arithmetic optimization method is constructed and used to optimize the extreme gradient boosting hyperparameters for COVID-19 chest X-ray pictures. To solve the problem of early convergence, this study introduces a novel variant known as the adaptive seagull optimization algorithm. The performance of the suggested algorithm is improved by increasing the seagulls' inclination towards exploratory behaviour^93^. Qusai-random sequences are employed for the population initialization in place of a random distribution in order to increase the variety and convergence factors. The authors of Ref.^94^ proposed an enhanced PSO algorithm that uses pseudo-random sequences and opposing rank inertia weights instead of random distributions for initialization to improve convergence speed and population diversity. The authors also introduced a new initialization population approach that uses a quasi-random sequence to initialize the swarm and generates an opposing swarm using the opposition-based method. For fifteen UCI data sets, the suggested technique optimized feed-forward neural network weight.

## Research gaps

The GWO is one of the well-known metaheuristic algorithms^95^. This algorithm draws inspiration from the hunting behaviour of grey wolves and the hierarchical leadership model. The GWO has been implemented, and the results have been encouraging enough to warrant additional research. Investigators can improve the issues of low precision and slow convergence speed^96^. As a result, studies utilized various techniques to increase optimizers' efficiency and address optimization issues. For instance, an improved GWO was suggested to adjust the recurrent neural network's parameters. To allow faster GWO convergence, chaotic GWO was introduced. Researchers have also employed numerous techniques to enhance GWO^97–99^. The literature review has been extensively augmented to underscore the existing research gaps within the context of optimization algorithms applied to data clustering, with a specific focus on the limitations of current metaheuristic algorithms, including the traditional GWO. Despite GWO's proven effectiveness in various optimization tasks, its application to data clustering reveals critical shortcomings, primarily its struggle with premature convergence and its inability to maintain a balance between exploration and exploitation phases. These limitations significantly affect the quality of clustering outcomes, especially in complex datasets with high dimensionality or noise. Moreover, while existing studies have explored numerous enhancements to GWO and other metaheuristic algorithms, there remains a distinct gap in the literature regarding the integration of these algorithms with classical clustering techniques, such as K-means, to address these specific challenges. This gap highlights the need for innovative approaches that can leverage the strengths of both metaheuristic optimization and traditional clustering methods to achieve superior clustering performance. The proposed $K$-means Clustering-based Grey Wolf Optimizer (KCGWO) aims to fill this gap by introducing a hybrid algorithm that combines the adaptive capabilities of GWO with the efficiency of K-means clustering. This combination is designed to enhance diversity, prevent premature convergence, and ensure a more effective balance between the exploration of new solutions and the exploitation of known good solutions. However, the literature review reveals that while there are various attempts to improve the clustering process through algorithmic enhancements, the specific approach of blending GWO with K-means, complemented by a dynamic weight factor to adjust exploration and exploitation dynamically, is notably absent. This research gap signifies an opportunity for the KCGWO to contribute significantly to the field, offering a novel solution that addresses both the limitations of traditional GWO in clustering tasks and the need for more effective hybrid algorithms. By clarifying these gaps and positioning the KCGWO within this context, the revised related works section establishes a strong foundation for the significance and novelty of the proposed research.

### Need for the proposed algorithm

In addition to the metaheuristic algorithm, the primary goal of the data mining process is to gather data from a big data set. The data can then be translated into a clear format for further usage. Clustering is a popular experimental data analysis tool. Objects are arranged using clustering so that each cluster contains more comparable objects. As discussed earlier, various cluster methods have been created to group the data. $K$-means is an example of a partitioning clustering algorithm because it operates based on the cluster centroid^15,19^. Numerous uses of the $K$-means cluster have been documented. In addition to enhancing the reliability of wireless sensor networks, $K$-means clustering was also used for image segmentation. Additionally, as an unsupervised learning technique, the $K$-means cluster has been frequently utilized to categorize data with no labels. The primary objective is to propose another variant of GWO called KCGWO to solve complex optimization problems, including data clustering problems. In this study, the KCGWO is proposed as an advanced solution to the inherent limitations of the GWO in addressing data clustering challenges. The GWO, while innovative in mimicking the social hierarchy and hunting tactics of grey wolves, exhibits deficiencies in exploration and exploitation—key factors for effective clustering. The proposed KCGWO method enhances GWO by incorporating the K-means algorithm and introducing a dynamic weight factor, aiming to improve the algorithm's performance significantly. The methodology of KCGWO unfolds in two pivotal enhancements over the traditional GWO. First, the integration of the K-means algorithm serves as an initial refinement step. Before the optimization process, K-means is applied to the dataset to establish a preliminary grouping of data points. This step ensures that the starting positions of the grey wolves (solutions) are closer to potential optimal solutions, thereby enhancing the exploration phase of GWO. The initial clustering helps in guiding the wolves towards promising areas of the search space from the onset. Second, a dynamic weight factor is introduced to adjust the influence of exploration and exploitation dynamically throughout the optimization process. This weight factor varies the wolves' movements, allowing for a more flexible search strategy that can adapt based on the current state of the search. It enables the algorithm to maintain a balance between exploring new areas and exploiting known promising regions, thus preventing premature convergence to suboptimal solutions. The performance of KCGWO was evaluated through extensive testing on numerical benchmarks and real-world datasets, demonstrating its superior capability to efficiently navigate the solution space and locate optimal cluster centers swiftly. This effectiveness is attributed to the synergistic combination of K-means for initial solution enhancement and the dynamic weight factor for maintaining an optimal balance between exploration and exploitation. Overall, KCGWO represents a significant advancement in solving data clustering problems, offering a robust and reliable method that overcomes the limitations of GWO. Its innovative approach to integrating K-means with a dynamic adjustment mechanism ensures high-quality solutions, making it a valuable tool for data analytics and clustering applications.

The primary contributions of this study are discussed as follows:

- A new variant of GWO called KCGWO based on $K$-means clustering algorithm and weight factors in the position update is proposed.
- Formulation fitness function for data clustering problem of a machine learning systems.
- The performance of the KCGWO is validated using 10 numerical test functions and data clustering problems using eight real-world data sets with different dimensions.
- The performance comparison is made with other well-known algorithms based on the statistical data analysis and statistical Friedman's ranking test (FRT).

The paper has been structured as follows. Section "Data clustering and problem statement" discusses the data clustering concepts and the formulation of the fitness function for the same problem. Section "Proposed K-means clustering grey wolf optimizer" comprehensively presents the formulation of the proposed KCGWO based on the $K$-means clustering algorithm; in addition, the basic concepts of GWO are also discussed. The results are comprehensively discussed in Section "Results and discussions", and Sect. "Conclusions" concludes the paper with a future study.

## Data clustering and problem statement

The basic objective of data mining techniques is to obtain features from huge volumes of data. Such techniques use data processing techniques to find interesting patterns in huge amounts of data. Clustering, classifications, detecting anomalies, detecting deviations, synthesizing, and regression are a few examples of data analysis techniques. Data clustering is dividing a set of information into smaller groups where the similarities between the individuals in each group are high while those between the data in other groups are low. Distance metrics like Euclidean distance, Chord distance, and Jaccard index are used to assess how similar individuals of a subset are to one another. In principle, clustering algorithms can be divided into two groups: partitional and hierarchical, depending on how clusters are created and maintained^86^. A tree that depicts a sequence of clusters is produced in hierarchical clustering with no background knowledge of the number of groups or dependence on the initial state. Nevertheless, because they are static, an entity allocated to one cluster cannot be moved to another. Hierarchical algorithms' main drawback is this. The incompetent clustering of overlapping clusters could also be due to a lack of planning regarding the number of clusters. On the other hand, partitional clustering divides items into clusters of a predetermined size. Various techniques for partitional clustering aim to increase the dissimilarity of members belonging to distinct clusters while attempting to reduce the difference between objects in each cluster^27,100–102^.

Typically, the Euclidian distance is used to measure similarity. In this work, the distance between any two objects (${o}_{i}$ and ${o}_{j}$) inside the cluster is also determined using the Euclidean distance measure. Typically, it could be expressed as follows^103^:

1 $$D\left({o}_{i},{o}_{j}\right)=\parallel {o}_{i}-{o}_{j}\parallel =\sqrt{\sum_{m=1}^{d}{\left({o}_{im}-{o}_{jm}\right)}^{2}} ,$$

where ${o}_{i}$ and ${o}_{j}$ denote two distinct objects inside the cluster, $d$ denotes the number of features for the entity, and partition clustering can be transformed into an optimization model based on the similarity metric, and this model can be explained as follows:

2 $$\underset{Z,W}{{\text{Minimize}}}: f\left(Z,W\right)=\sum_{k=1}^{K}\sum_{i=1}^{n}{w}_{ik}D\left({x}_{i},{z}_{k}\right) ,$$

Subjected to:

3 $$\left\{\begin{array}{ccc}\sum_{k=1}^{K}{w}_{ik}=1,& i=\mathrm{1,2},\dots ,n;& \\ {w}_{ik}\in \left\{\mathrm{0,1}\right\},& \forall i\in \left\{\mathrm{1,2},\dots ,n\right\},k\in \left\{\mathrm{1,2},\dots ,K\right\}& \end{array}\right.$$

where $n$ denotes the sample size, $K$ denotes the cluster size, and ${x}_{i}$ signifies the coordinates of the $i$ th object in the current datasets. The term ${w}_{ik}$ indicates whether the $i$ th object is clustered into the $k$ th cluster or not, and $D\left({x}_{i},{z}_{k}\right)$ indicates the length between the $i$ th object and the center of the $k$ th cluster. Noteworthy is the fact that the following is used to observe the same.

4 $$W=\{{w}_{ik}|i=\mathrm{1,2},\dots ,n,k=\mathrm{1,2},\dots ,K$$

A sample's partition criteria determine the amount of ${w}_{ik}$ in Eq. (3). Obtain an object partition that meets Eq. (3) for given a sample set $X=\left\{{x}_{1},{x}_{2}, \dots ,{x}_{n}\right\}$.

5 $$\left\{\begin{array}{ccc}\sum_{i=1}^{K}{C}_{i}=X;& & \\ {C}_{i}\cap {C}_{j}=\phi ,& \forall i,j\in \left\{\mathrm{1,2},\dots ,K\right\}\wedge i\ne j;& \\ {C}_{i}\ne \phi ,& \forall i\in \left\{\mathrm{1,2},\dots ,K\right\}& \end{array}\right.$$

where ${C}_{i}(i=\mathrm{1,2},\dots ,K)$ is the $i$ th cluster's object set, and the following equation can be used to identify its members:

6 $$\left\{\begin{array}{ccc}{C}_{i}=\left\{{x}_{k}|\parallel {x}_{k}-{z}_{i}\parallel \le \parallel {x}_{k}-{z}_{p}\parallel ,{x}_{k}\in X\right\},& p\ne i,p=\mathrm{1,2},\dots ,K;& \\ {z}_{i}=\frac{1}{\left|{C}_{i}\right|}\sum_{{x}_{k}\in {C}_{i}}{x}_{k},& i=\mathrm{1,2},\dots ,K& \end{array}\right.$$

where ${z}_{i}$ is frequently employed in the $k$-means clustering method, symbolizes a new center of cluster $i,$ and $\parallel .\parallel$ indicates the Euclidean distance between any two items in the subset.

## Proposed K-means clustering-based grey wolf optimizer

This section briefs the original concepts of the basic Grey Wolf Optimizer (GWO) with its mathematical modelling. The proposed K-means Clustering-based Grey Wolf Optimizer (KCGWO) is discussed comprehensively.

### Grey wolf optimizer

The grey wolf optimization algorithm is the most contemporary breakthrough in the field of metaheuristic optimization and was initially devised in Ref.^95^. GWO mimics the hunting actions of grey wolves in the wild, a supporting approach they use to chase their prey. The framework of the GWO seems quite distinct compared to other meta-heuristic optimization in that it uses three optimal specimens as the basis for a complex search procedure. These three optimal specimens are an alpha wolf $\alpha$ that serves as the pack leader, a beta wolf $\beta$ that provides support to the leader, and a delta wolf $\delta$ that follows the leader and the loyal wolves. The last kind of wolf is termed omega wolf $\omega$. Such wolves have varying degrees of responsibility and can be arranged in a hierarchy, with $\alpha$ being the highest level and the first solution, $\beta$, $\delta$, and $\omega$ representing the second, third, and final solutions, correspondingly. Thus, the three wolves mentioned above serve as inspiration for omegas. All species of wolves employ the three separate coefficients utilized to implement the encircling process to attempt to encompass the prey when they have located it. Three wolves evaluate the potential location of prey during the iterative search strategy. Based on Eqs. (7), (8), the positions of the wolf are updated during the optimization procedure.

7 $$\overrightarrow{D}=\left|\overrightarrow{C}\cdot \overrightarrow{{X}_{P}}\left(t\right)-\overrightarrow{X}\left(t\right)\right| ,$$

8 $$\overrightarrow{X}\left(t+1\right)=\overrightarrow{{X}_{P}}\left(t\right)-\overrightarrow{A}\cdot \overrightarrow{D} ,$$

where $t$ is the current iteration, $\overrightarrow{C}$ and $\overrightarrow{A}$ are coefficient vectors, $\overrightarrow{{X}_{P}}$ signifies the prey's position, and $\overrightarrow{X}$ signifies the wolf position. The vectors $\overrightarrow{C}$ and $\overrightarrow{A}$ are as follows:

9 $$\overrightarrow{A}=2a\cdot \overrightarrow{{r}_{1}}-\overrightarrow{a} ,$$

10 $$\overrightarrow{C}=2\cdot \overrightarrow{{r}_{2}} ,$$

where $\overrightarrow{{r}_{1}}$ and $\overrightarrow{{r}_{2}}$ signify random vectors in the range $[0, 1]$, and factor $a$ linearly falls from 2 to 0 with the number of iterations. The wolf at a location can change its position about the prey using the aforementioned updating algorithms. By changing the random parameters $\overrightarrow{A}$ and $\overrightarrow{C}$, it may be made to move to any location in the continuous space close to prey. The GWO considers that the prey's position is likely in the alpha, beta, and delta positions. During searching, the best, second-best, and third-best individuals found so far are recorded as alpha, beta, and delta. Omega wolves, on the other hand, change their sites in accordance with alpha, beta, and delta wolf populations.

11 $$\left.\begin{array}{c}{\overrightarrow{D}}_{\alpha }=\left|\overrightarrow{{C}_{1}}\cdot \overrightarrow{{X}_{\alpha }}-\overrightarrow{X}\right|\\ {\overrightarrow{D}}_{\beta }=\left|\overrightarrow{{C}_{2}}\cdot \overrightarrow{{X}_{\beta }}-\overrightarrow{X}\right|\\ {\overrightarrow{D}}_{\delta }=\left|\overrightarrow{{C}_{3}}\cdot \overrightarrow{{X}_{\delta }}-\overrightarrow{X}\right|\end{array}\right\} .$$

The location vectors for $\alpha$, $\beta$, and $\delta$ are, respectively, $\overrightarrow{{X}_{\alpha }}$, $\overrightarrow{{X}_{\beta }}$, and $\overrightarrow{{X}_{\delta }}$. The vectors $\overrightarrow{{C}_{1}}$, $\overrightarrow{{C}_{2}}$, and $\overrightarrow{{C}_{3}}$ were produced randomly, and $\overrightarrow{X}$ indicates the current position vector. The distances between the position of the current person and those of alpha, beta, and delta are calculated, respectively, by Eq. (11). To determine the present person's final position matrices, the following are described.

12 $$\left.\begin{array}{c}\overrightarrow{{X}_{1}}=\overrightarrow{{X}_{\alpha }}-\overrightarrow{{A}_{1}}\cdot \left(\overrightarrow{{D}_{\alpha }}\right)\\ \overrightarrow{{X}_{2}}=\overrightarrow{{X}_{\beta }}-\overrightarrow{{A}_{2}}\cdot \left(\overrightarrow{{D}_{\beta }}\right)\\ \overrightarrow{{X}_{3}}=\overrightarrow{{X}_{\delta }}-\overrightarrow{{A}_{3}}\cdot \left(\overrightarrow{{D}_{\delta }}\right)\end{array}\right\} ,$$

13 $$\overrightarrow{X}\left(t+1\right)=\frac{\overrightarrow{{X}_{1}}+\overrightarrow{{X}_{2}}+\overrightarrow{{X}_{3}}}{3} ,$$

where $\overrightarrow{{A}_{1}}$, $\overrightarrow{{A}_{2}}$, and $\overrightarrow{{A}_{3}}$ denote vectors that are randomly created, and $t$ signifies the current iteration. The regulating factor that modifies the coefficient $\overrightarrow{A}$ is variable $a$. This tactic aids the population in deciding whether to pursue or flee its prey. As a result, if $|A|$ has a value greater than 1, the wolf is trying to find new search spaces. However, the wolf could pursue and attack the prey if the value of $|A|$ is smaller than 1. The grey wolf starts to prevent any motion of the prey from attacking it once the hunting is accomplished adequately. This technique is accomplished by lowering the value of $a$, which is in the range of 2 and 0. The value of an also decreases the value of $\overrightarrow{A}$, which now falls between [− 1, 1]. The pseudocode of the GWO is provided in Algorithm 1.

**Algorithm 1:**
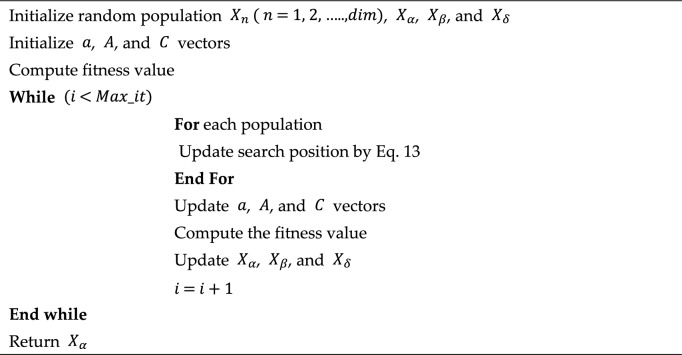
Pseudocode of grey wolf optimizer.

### K-means clustering-based grey wolf optimizer

In addition to the significance of optimization techniques, data analysis is a key research area. Clustering has thus been utilized as one of the data exploration approaches to gain a general understanding of the data's architecture. $K$-means is the most used unsupervised algorithm. Data points in each group resemble each other much more than those in other clusters. The method does a sequence of operations to identify unique subsets, which are discussed below.

- The number of subsets is the primary criterion for the K-means, which in data mining starts with the initial set of random centroids chosen for each cluster.
- The following step involves determining the Euclidean distance from the center to every data point in specific information set to connect each data with the closest point.
- Continue performing until there is no modification in the centroids if K centres shift during the iteration.

The algorithm attempts to minimize the squared error function or objective function presented in Eq. (14).

14 $$\sum_{i=1}^{C}\sum_{j=1}^{{C}_{i}}{\Vert {x}_{i}^{j}-{C}_{j}\Vert }^{2} ,$$

where ${x}_{i}^{j}$ signifies the data points of $i$ th cluster, ${C}_{j}$ denotes the size of the cluster center, and $\Vert {x}_{i}^{j}-{C}_{j}\Vert$ represents the Euclidean distance between ${C}_{j}$ and ${x}_{i}^{j}$. The initialization of the proposed KCGWO is similar to the original version of GWO. $K$-means is utilized to separate the grey wolf population into three groups. The objective function value is then determined for each cluster/population individually^104^. The population has been divided into three clusters based on a random integer. If the random number is greater than 0.5, KCGWO uses population clusters based on the fitness values of each cluster. All the clusters' fitness values are compared within the condition. The population position is equal to cluster position 1, position 2, or position 3 based on the conditions provided in the pseudocode. However, KCGWO operates on the actual population without clustering if the random value is less than or equal to 0.5. Therefore, this feature can be utilized with different methods, but it needs to be evaluated to ensure it functions well. However, $K$-means are utilized in this study to enhance the effectiveness of GWO. The proposed KCGWO tries to compute the fitness for each population after selecting a particular population with/without clustering until it discovers the best fitness. Equations (7)–(12) determine the optimum search agents. Equation (13) is then used to update each position. However, the weightage is not provided for the wolf hierarchy. Therefore, in Eq. (13), weight factors are introduced to improve the solution quality^105^. The modified position update equation is provided in Eq. (15).

15 $$\overrightarrow{X}\left(t+1\right)=\frac{3\overrightarrow{{X}_{1}}+\overrightarrow{2{X}_{2}}+\overrightarrow{{X}_{3}}}{6} ,$$

**Figure 1:**
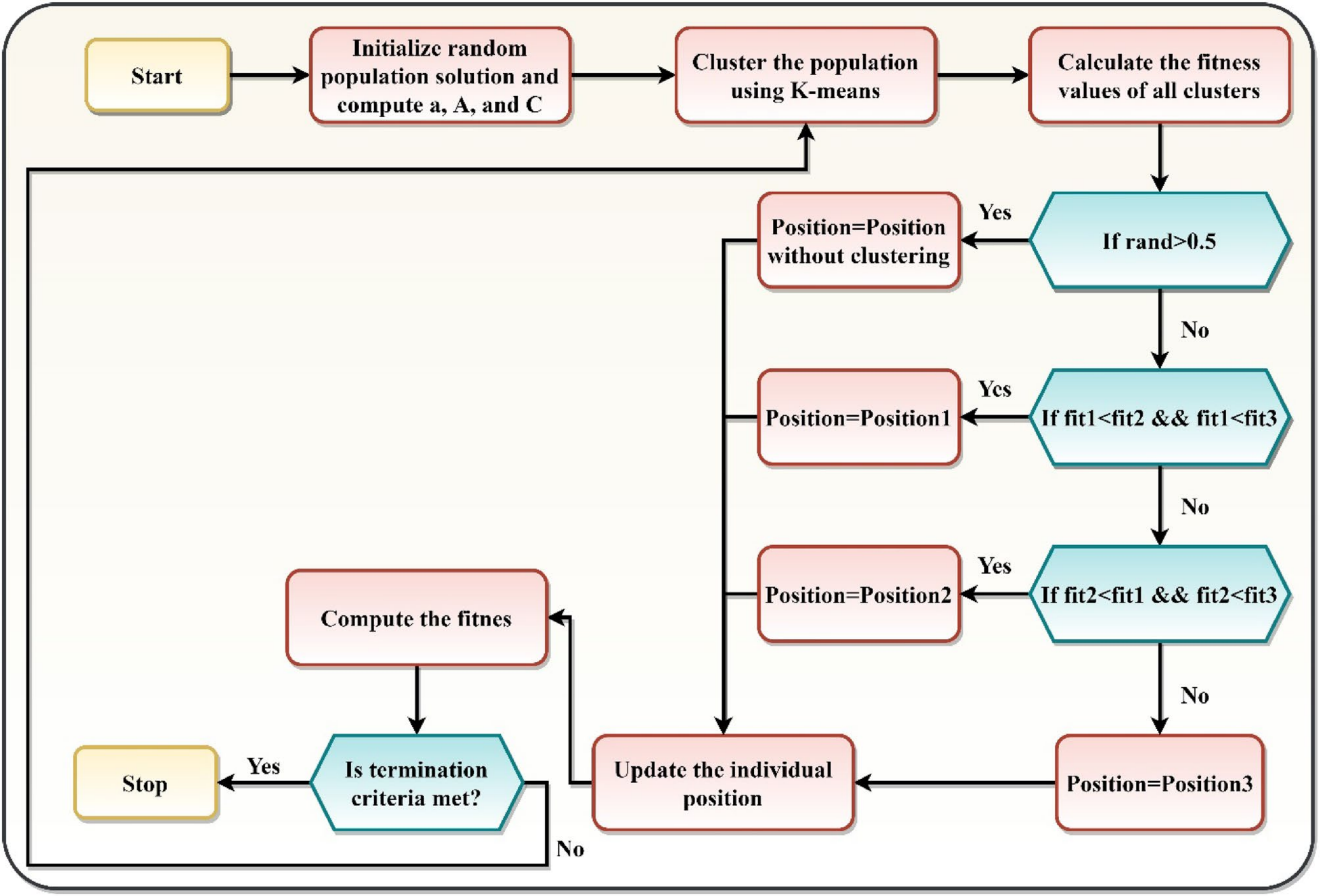
Flowchart of the proposed algorithm.

The variables $a$, $\overrightarrow{A}$ and $\overrightarrow{C}$ are updated for the subsequent iteration. As a result, the iteration's best fit is chosen. Finally, the best fitness and position are returned. Figure 1 illustrates the flowchart of the proposed KCGWO algorithm. Algorithm 2 depicts the pseudocode of the KMGWO algorithm.

### Application of the proposed KCGWO to data clustering

A crucial stage in every metaheuristic approach is solution encoding. Each solution (grey wolf) represents all the cluster centers. These solutions are first produced randomly. However, the best position at each iteration of the KCGWO serves as a guide for the remaining grey wolves.

**Figure 2:**
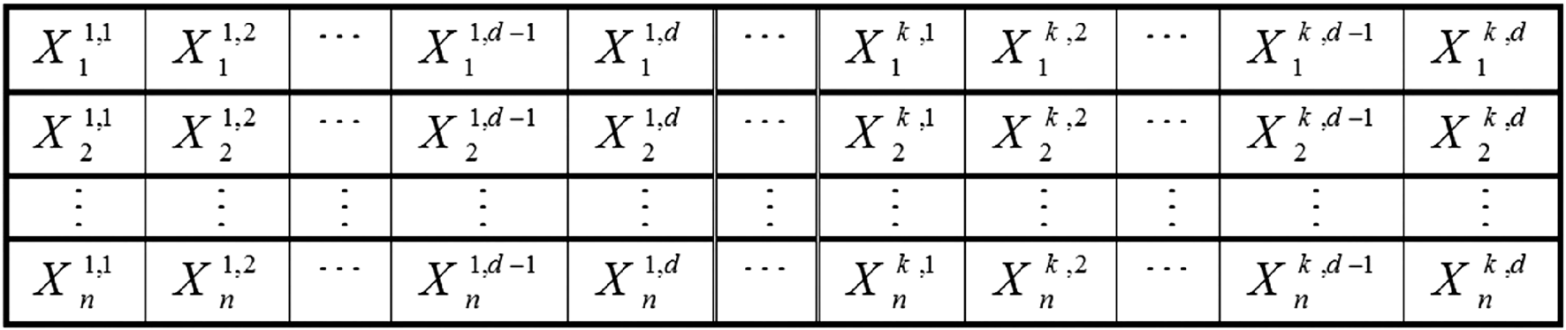
Illustration of solution encoding^96^.

Each answer is an array of size $d\times k$, with $d$ being the total number of characteristics for each data and $k$ being the total clusters. Figure 2 displays a pack of grey wolves representing the solutions. The fitness function is the total intra-cluster distance. The fitness function must be minimized to discover the best cluster centers using KCGWO. It is preferred to reduce the sum of intra-cluster distances^96^. In Eq. (16), the cluster center is defined, and Eq. (17) defines the distances between cluster members.

16 $${Y}_{j}=\frac{1}{{n}_{j}}\sum_{\forall {x}_{p}\in {C}_{j}}{x}_{p} ,$$

17 $${\text{Distance}}\left({x}_{p}-{y}_{j}\right)=\sqrt{\sum_{i=1}^{a}{\left({x}_{p}-{y}_{j}\right)}^{2}}$$

where ${y}_{j}$ denotes cluster center, ${x}_{p}$ denotes the position of the $p$ th cluster member, $a$ denotes the number of features of the dataset, ${n}_{j}$ denotes the members in the cluster $j$, and ${C}_{j}$ denotes the cluster member $j$.

**Algorithm 2:**
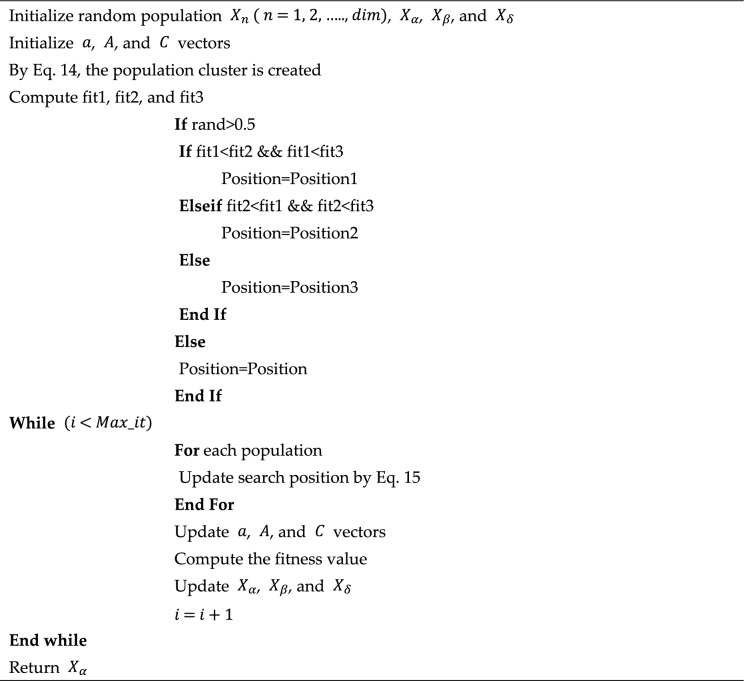
Pseudocode of the proposed KCGWO.

### Computational complexity

The computational complexity of the KCGWO is discussed as follows: (i) The proposed algorithm necessitates $O(N\hspace{0.17em}\times \hspace{0.17em}dim)$, where $N$ denotes the number of search agents, i.e. population size and $dim$ denotes the problem dimension, (ii) the control parameters of KCGWO necessitates $O(N\hspace{0.17em}\times \hspace{0.17em}dim)$, (iii) the position update of the KCGWO necessitates $O(N\hspace{0.17em}\times \hspace{0.17em}dim)$, and (iv) fitness values of each population and cluster necessitate $O(N\hspace{0.17em}\times \hspace{0.17em}dim\times n)$, where $n$ denotes the number of clustered population. Based on discussions, the complexity of KCGWO for each iteration is $O(N\hspace{0.17em}\times \hspace{0.17em}dim\times n)$, and finally, the total complexity of the proposed KCGWO algorithm is $O(N\hspace{0.17em}\times \hspace{0.17em}dim\times n\times Max\_it)$, where $Max\_it$ denotes the maximum of iterations.

## Results and discussions

The original GWO is improved by employing the K-means clustering concept along with the weight factor, and it has been tested using 10 benchmark numerical functions, which have both unimodal and multimodal features. In addition, the performance is also validate for data clustering problems. The performance of the proposed KCGWO is compared with four other algorithms, such as MFO, SSA, MVO, ASO, PSO, JAYA, and the original GWO algorithm. The population size is 30, and the maximum number of iterations is 500 for all selected algorithms. All the algorithms are implemented using MATLAB software installed on a laptop with an i5 processor, a 4.44 GHz clock frequency, and 16 GB of memory. The algorithms are executed 30 times individually for a fair comparison.

### Results for numerical optimization functions

The details of the selected benchmark functions are recorded in Table 2. The functions F1-F4 have unimodal features with 30 dimensions, F5-F7 have multimodal features with 30 problem dimensions, & F8-F10 have multimodal features with very low dimensions. The purpose of selecting the listed benchmark function is to analyze the exploration and exploitation behaviour of the developed KCGWO algorithm. The statistical measures, such as minimum (Min), Mean, maximum (Max), and Standard Deviation (STD) of all designated algorithms, are recorded in Table 3.

**Table 2:** 10 benchmark test functions for validation.

Function	Dim	Range	*f*_min_
${f}_{1}\left(x\right)={\sum }_{i=1}^{n} {x}_{i}^{2}$	30	[− 100, 100]	0
${f}_{2}\left(x\right)={\sum }_{i=1}^{n} \left|{x}_{i}\right|+{\prod }_{i=1}^{n} \left|{x}_{i}\right|$	30	[− 10, 10]	0
${f}_{3}\left(x\right)={\sum }_{i=1}^{n} {\left({\sum }_{j-1}^{i} {x}_{j}\right)}^{2}$	30	[− 100, 100]	0
${f}_{4}\left(x\right)={{\text{max}}}_{i}\left\{\left|{x}_{i}\right|\text{,}1\le i\le n\right\}$	30	[− 100, 100]	0
${f}_{5}(x)={\sum }_{i=1}^{n} [{x}_{i}^{2}-10{\text{cos}}(2\pi {x}_{i})+10]$	30	[− 5.12, 5.12]	0
${f}_{6}(x)=-20{\text{exp}}\left(-0.2\sqrt{\frac{1}{n}{\sum }_{i=1}^{n} {x}_{i}^{2}}\right)-{\text{exp}}\left(\frac{1}{n}{\sum }_{i=1}^{n} {\text{cos}}(2\pi {x}_{i})\right)+20+e$	30	[− 32, 32]	0
${f}_{7}(x)=\frac{1}{4000}{\sum }_{i=1}^{n} {x}_{i}^{2}-{\prod }_{i=1}^{n} {\text{cos}}\left(\frac{{x}_{i}}{\sqrt{i}}\right)+1$	30	[− 600, 600]	0
${f}_{8}(x)=4{x}_{1}^{2}-2.1{x}_{1}^{4}+\frac{1}{3}{x}_{1}^{6}+{x}_{1}{x}_{2}-4{x}_{2}^{2}+4{x}_{2}^{4}$	2	[− 5, 5]	− 1.0316
${f}_{9}(x)={\left({x}_{2}-\frac{5.1}{4{\pi }^{2}}{x}_{1}^{2}+\frac{5}{\pi }{x}_{1}-6\right)}^{2}+10\left(1-\frac{1}{8\pi }\right){\text{cos}}{x}_{1}+10$	2	[− 5, 5]	0.398
${f}_{10}(x)=-{\sum }_{i=1}^{10} {[(X-{a}_{i}){(X-{a}_{i})}^{T}+{c}_{i}]}^{-1}$	4	[0, 10]	− 10.5363

**Table 3:** Results obtained for 10 benchmark functions by all selected algorithms.

Functions	Metrics	KCGWO	GWO	MFO	MVO	SSA	JAYA	AOS	PSO
${f}_{1}$	Min	0.00E + 00	2.65E-29	3.00E + 02	8.70E-01	3.41E-08	0.887679	2.84E-80	899.5347
	Max	1.74E-158	8.74E-28	5.42E + 02	1.73E + 00	6.13E-07	72.8437	3.79E-76	11,137.22
	Mean	*0.00E* + *00*	1.82E-28	3.24E + 02	1.70E + 00	1.90E-07	1.454592	3.81E-80	1032.142
	STD	1.01E-158	4.51E-28	1.33E + 02	4.88E-01	3.00E-07	0.234375	3.859375	0.052083
	Rank	**1**	3	7	5	4	6	2	8
${f}_{2}$	Min	0.00E + 00	9.51E-18	1.48E + 01	5.95E-01	1.35E + 00	1.17E-02	7.67E-55	1.67E + 01
	Max	0.00E + 00	2.98E-16	2.63E + 01	8.89E-01	4.58E + 00	1.75E-01	3.73E-53	4.30E + 01
	Mean	*0.00E* + *00*	6.38E-17	2.42E + 01	6.93E-01	3.00E + 00	1.02E-01	1.72E-53	2.40E + 01
	STD	0.00E + 00	1.54E-16	6.11E + 00	1.50E-01	1.61E + 00	9.90E-02	3.17E + 00	4.17E-02
	Rank	**1**	3	8	5	6	4	2	7
${f}_{3}$	Min	2.65E-99	3.85E-07	1.38E + 04	1.66E + 02	6.98E + 02	9.35E + 03	2.85E + 04	7.62E + 03
	Max	9.44E-78	8.42E-04	4.61E + 04	2.19E + 02	2.26E + 03	1.34E + 04	5.31E + 04	3.98E + 04
	Mean	*4.84E-96*	5.16E-05	2.17E + 04	1.76E + 02	1.02E + 03	1.06E + 04	3.20E + 04	3.51E + 04
	STD	5.45E-78	4.72E-04	1.68E + 04	2.83E + 01	8.22E + 02	1.51E-01	3.34E + 00	1.20E-01
	Rank	**1**	2	6	3	4	5	7	8
${f}_{4}$	Min	0.00E + 00	2.02E-07	5.19E + 01	1.09E + 00	8.02E + 00	1.65E + 01	1.07E + 01	6.90E + 01
	Max	2.96E-84	6.98E-07	7.18E + 01	2.13E + 00	1.36E + 01	3.80E + 01	7.70E + 01	7.50E + 01
	Mean	*3.50E-87*	2.22E-07	6.58E + 01	1.74E + 00	9.02E + 00	1.70E + 01	3.58E + 01	7.16E + 01
	STD	1.71E-84	2.81E-07	1.02E + 01	5.27E-01	2.97E + 00	5.73E-02	3.16E + 00	5.21E-02
	Rank	**1**	2	7	3	4	5	6	8
${f}_{5}$	Min	0.00E + 00	9.66E-13	1.16E + 02	8.15E + 01	4.48E + 01	1.25E + 00	0.00E + 00	1.32E + 02
	Max	0.00E + 00	5.40E + 00	1.41E + 02	1.30E + 02	5.77E + 01	1.26E + 01	0.00E + 00	1.75E + 02
	Mean	*0.00E* + *00*	1.59E-12	1.36E + 02	1.26E + 02	4.58E + 01	7.20E + 00	0.00E + 00	1.63E + 02
	STD	0.00E + 00	3.12E + 00	1.32E + 01	2.70E + 01	7.20E + 00	8.85E-02	3.01E + 00	4.17E-02
	Rank	**1**	3	6	5	8	4	2	7
${f}_{6}$	Min	8.88E-16	1.00E-13	2.00E + 01	1.10E + 00	1.78E + 00	2.02E + 01	4.44E-15	2.00E + 01
	Max	8.88E-16	1.11E-13	2.00E + 01	2.16E + 00	3.35E + 00	2.02E + 01	4.44E-15	2.00E + 01
	Mean	*8.88E-16*	1.04E-13	2.00E + 01	1.21E + 00	2.89E + 00	2.02E + 01	4.44E-15	2.00E + 01
	STD	0.00E + 00	5.43E-15	5.34E-05	5.86E-01	8.06E-01	9.90E-02	2.91E + 00	5.73E-02
	Rank	**1**	3	6	4	5	8	2	7
${f}_{7}$	Min	0.00E + 00	0.00E + 00	6.98E + 00	8.38E-01	4.94E-03	1.03E + 00	0.00E + 00	5.85E + 00
	Max	0.00E + 00	1.23E-02	1.01E + 02	9.33E-01	1.96E-02	1.09E + 00	0.00E + 00	6.77E + 01
	Mean	*0.00E* + *00*	*0.00E* + *00*	4.36E + 01	8.42E-01	1.23E-02	1.08E + 00	0.00E + 00	6.28E + 00
	STD	0.00E + 00	7.12E-03	4.73E + 01	5.37E-02	7.32E-03	7.81E-02	2.86E + 00	6.25E-02
	Rank	**1**	2	8	5	4	6	3	7
${f}_{8}$	Min	− 1.03163	− 1.03163	− 1.03163	− 1.03163	− 1.03162	− 1.0316	− 1.0316	− 1.0316
	Max	− 1.03163	− 1.03163	− 1.03163	− 1.03163	− 1.03152	− 1.0316	− 1.0316	− 1.0316
	Mean	* − 1.03163*	* − 1.03163*	* − 1.03163*	* − 1.03163*	− 1.03162	− 1.0316	− 1.0316	− 1.0316
	STD	0	6.62E-08	0	6.21E-07	5.64E-05	1.93E-05	2.35E-10	1.57E-16
	Rank	**1**	5	**1**	6	8	7	4	3
${f}_{9}$	Min	0.397887	0.397888	0.397887	0.397887	0.39793	0.39823	0.39789	0.39789
	Max	0.397887	0.397898	0.397887	0.397888	0.399332	0.39918	0.39790	0.39789
	Mean	*0.397887*	0.397889	*0.397887*	*0.397887*	0.398836	0.39872	0.39789	0.39789
	STD	0	5.64E-06	0	9.32E-08	0.000711	4.75E-04	4.99E-06	0.00E + 00
	Rank	**1**	6	**1**	4	8	7	5	**1**
${f}_{10}$	Min	− 10.5364	− 10.5351	− 10.5364	− 10.5361	− 4.96056	− 4.7879	− 10.5248	− 3.8354
	Max	− 10.5364	− 10.532	− 2.80663	− 10.5355	− 2.91529	− 2.3747	− 3.8349	− 2.4217
	Mean	− *10.5364*	− 10.5341	− 10.5364	− 10.536	− 3.09438	− 2.7739	− 5.1264	− 3.8354
	STD	2.81E-15	0.001572	4.46279	0.00034	1.132679	1.29E + 00	3.55E + 00	8.16E-01
	Rank	**1**	4	3	2	7	8	5	6
Average rank		**1**	3.3	5.3	4.2	5.8	6	3.8	6.2

Significant values are in bold and italic.

Classifying functions F01-F04 as unimodal test scenarios with a single global best is appropriate. Such test sets can be used to look into the general exploitation potential of the proposed KCGWO approach. Findings of the proposed KCGWO and other approaches, as well as their Min, Max, Mean, and STD, are shown in Table 2. The associated tables' higher outcomes are noted. The optimization techniques are then ordered based on their average values. The average rank is also calculated to determine the approaches' overall ranking. All Tables include a summary of the findings of the statistical analysis. The best results are emphasized in bold face in all tables. For each unimodal function, individual ranking is provided to examine the performance of the proposed algorithm. The proposed algorithm stands first out of all selected algorithms for all four unimodal functions.

The F1–F04 shows that the KCGWO can arrive at capable making with a suitable exploitation capability. This seems to be due to the effectiveness with which the suggested K-means clustering concept and weight factors can boost the GWO's tendencies for exploration and exploitation. As a result, the mechanisms make the algorithm more likely to produce smaller fitness and higher stability index values. This tool helps explore new locations close to the recently discovered results. Because of this, it was found that the new algorithmic changes have improved how GWO handles unimodal test cases. Assessing the exploration potential using multimodal functions (F5–F10) is reasonable. Table 2 shows that KCGWO can investigate highly competitive solutions for the F5–F10 test scenarios. The KCGWO can produce optimal results for all test functions compared to other approaches. According to the results, KCGWO can outperform all selected algorithms in multimodal instances. Additionally, statistical analyses show that, in 95% of evaluations, KCGWO outcomes are superior to those of other approaches. Compared to GWO, the accuracy is increased based on the STD index.

In particular, when the objective problems (F5-F8) involve several local optima, KCGWO's outperformance demonstrates a sufficient explorative nature. This is due to the effectiveness with which the K-means clustering structure can boost the GWO's performance for exploration and exploitation. Lower stability index values can encourage wolves to make more exploratory jumps. This feature might be seen when KCGWO requires investigating previously unexplored regions of the issue landscape. The weight factors have helped GWO achieve a delicate balance between its local and global search inclinations. According to the findings, the recommended K-means searching steps increase the GWO's exploration capability. Additionally, the KCGWO's update mechanism can lessen the likelihood of the KCGWO entering local optima. The exploratory propensity of KCGWO is hence advantageous. The computational complexity of the proposed algorithm is assessed by recording the RunTime (RT). The RT values for each function by all selected algorithms are recorded in Table 4. The average values of RT are also provided, and based on the mean RT value, the original GWO has less RT value, and the RT value of KCGWO is slightly greater than the GWO, which is due to the fact that the introduction of the K-means clustering mechanism. At the same time, the weight factor does not impact the proposed algorithm's computational complexity.

**Table 4:** RT values of each test functions.

Functions	KCGWO	GWO	MFO	MVO	SSA	JAYA	AOS	PSO
${f}_{1}$	0.276	0.1875	0.3073	0.4948	0.1927	0.2844	3.8594	0.3521
${f}_{2}$	0.0833	0.0521	0.1198	0.1146	0.0885	0.1198	2.8490	0.1469
${f}_{3}$	0.1823	0.1771	0.1563	0.2188	0.1563	0.2292	0.2240	0.2552
${f}_{4}$	0.0833	0.0469	0.0625	0.099	0.0625	0.1667	0.1302	0.1042
${f}_{5}$	0.1094	0.0885	0.1094	0.1563	0.1927	0.1254	0.1356	0.1563
${f}_{6}$	0.0625	0.0469	0.0729	0.1198	0.1198	0.1667	0.1302	0.1042
${f}_{7}$	0.0781	0.0625	0.0833	0.1042	0.1458	0.1719	0.1927	0.2292
${f}_{8}$	0.0677	0.0781	0.1094	0.1615	0.0729	0.1347	0.1451	0.1289
${f}_{9}$	0.0365	0.0781	0.0677	0.125	0.0677	0.0598	0.0747	0.0909
${f}_{10}$	0.0521	0.026	0.0469	0.0677	0.0521	0.0789	0.1151	0.0994
Average RT	0.10312	0.08437	0.11355	0.16617	0.1151	0.15375	0.7856	0.16673

**Figure 3:**
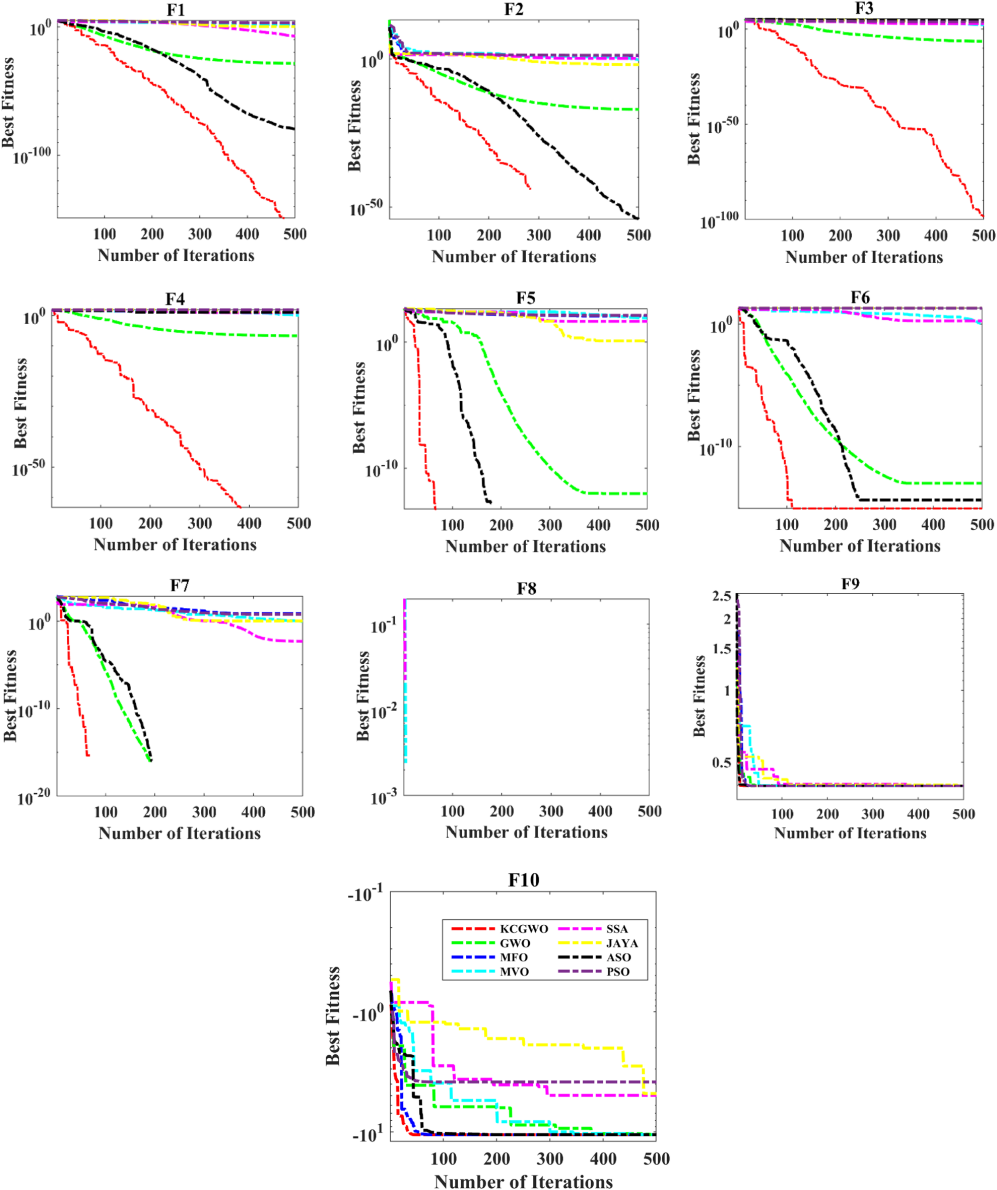
Convergence curve obtained by all algorithms.

**Figure 4:**
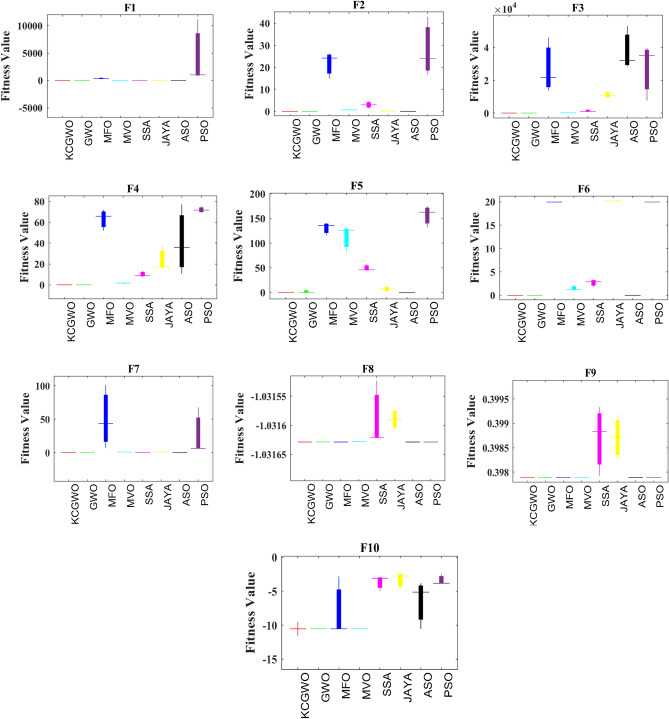
Boxplot analysis of all selected algorithms.

Figure 3 shows all selected algorithms' convergence characteristics for handling F1-F10 functions. All selected algorithms consistently outperform for a benchmark and have excellent convergence outfits in the original publication. Figure 3 also offers a convergence timeframe. It pinpoints the times when KCGWO performs better than GWO. According to Fig. 3, KCGWO eventually converges to superior outcomes. A large number of iterations allows KCGWO to approximate more precise solutions close to the optimum solutions. Additionally, rapid convergence patterns may be seen when comparing the curves of KCGWO and its original version. This pattern demonstrates that KCGWO can emphasize more exploitation and local search in the latter stages. These plots suggest that the KCGWO can successfully increase all wolves' fitness and promise to exploit improved results. In order to visualize the stability analysis, the boxplots are also plotted and shown in Fig. 4. From Fig. 4, it is detected that the stability of the KCGWO is better than all selected algorithms.

To further asses the performance of the proposed algorithm, the statistical non-parametric test, Friedman's Ranking Test (FRT), has been conducted, and the average FRT values of all algorithms are logged in Table 5. Based on the observation, the proposed algorithm attains the top of the table with an average FRT of 1.383, followed by GWO, AOS, MVO, SSA, MFO, JAYA, and PSO.

**Table 5:** FRT values of all algorithms for each test functions.

Functions	KCGWO	GWO	MFO	MVO	SSA	JAYA	AOS	PSO
${f}_{1}$	**1.000**	3.000	7.000	5.667	4.000	5.333	2.000	8.000
${f}_{2}$	**1.000**	3.000	7.667	5.000	6.000	4.000	2.000	7.333
${f}_{3}$	**1.000**	2.000	7.000	3.000	4.000	5.333	7.333	6.333
${f}_{4}$	**1.000**	2.000	7.000	3.000	4.000	5.333	6.333	7.333
${f}_{5}$	**1.000**	2.333	7.333	4.667	4.333	6.000	2.667	7.667
${f}_{6}$	1.667	4.333	7.333	4.667	**1.333**	6.000	3.000	7.667
${f}_{7}$	**2.000**	**2.000**	7.667	4.667	6.000	4.333	**2.000**	7.333
${f}_{8}$	**1.833**	5.000	**1.833**	6.000	7.333	7.667	4.000	2.333
${f}_{9}$	**2.000**	5.333	**2.000**	4.333	7.667	7.333	5.333	**2.000**
${f}_{10}$	**1.333**	3.667	3.000	2.667	6.000	7.333	5.333	6.667
Average FRT	**1.383**	3.267	5.783	4.367	5.067	5.867	4.000	6.267

Significant values are in bold.

These statistics indicate that the K-mean clustering approach and modified position update equation based on the weight factors can enhance the search functionality of GWO. Comparing the suggested KCGWO to existing approaches with superior convergence characteristics can be more effective.

### Results for data clustering problems

The suggested clustering approach was thoroughly assessed using eight datasets. Few datasets used are synthetic, and others are drawn from real-time benchmark data. Table 6 provides a summary of the traits of these datasets^106^. The features (dimensions), the total number of samples, and the number of clusters in each dataset are recorded in Table 6. The type of the problems is also mentioned. The dataset is selected based on the type and number of samples.

**Table 6:** Details of the selected dataset^106^.

Datasets	Dimensions	Samples	Cluster	Type
Emission	4	7384	6	Real-world
HTRU2	4	17,898	6	Artificial
Wine	14	178	2	Artificial
Breast cancer	30	569	2	Real-world
Sonar	60	208	2	Artificial
WDBC	30	568	2	Real-world
Iris	4	150	3	Real-world
2022 Ukraine Russia war	11	194	4	Real-world

**Figure 5:**
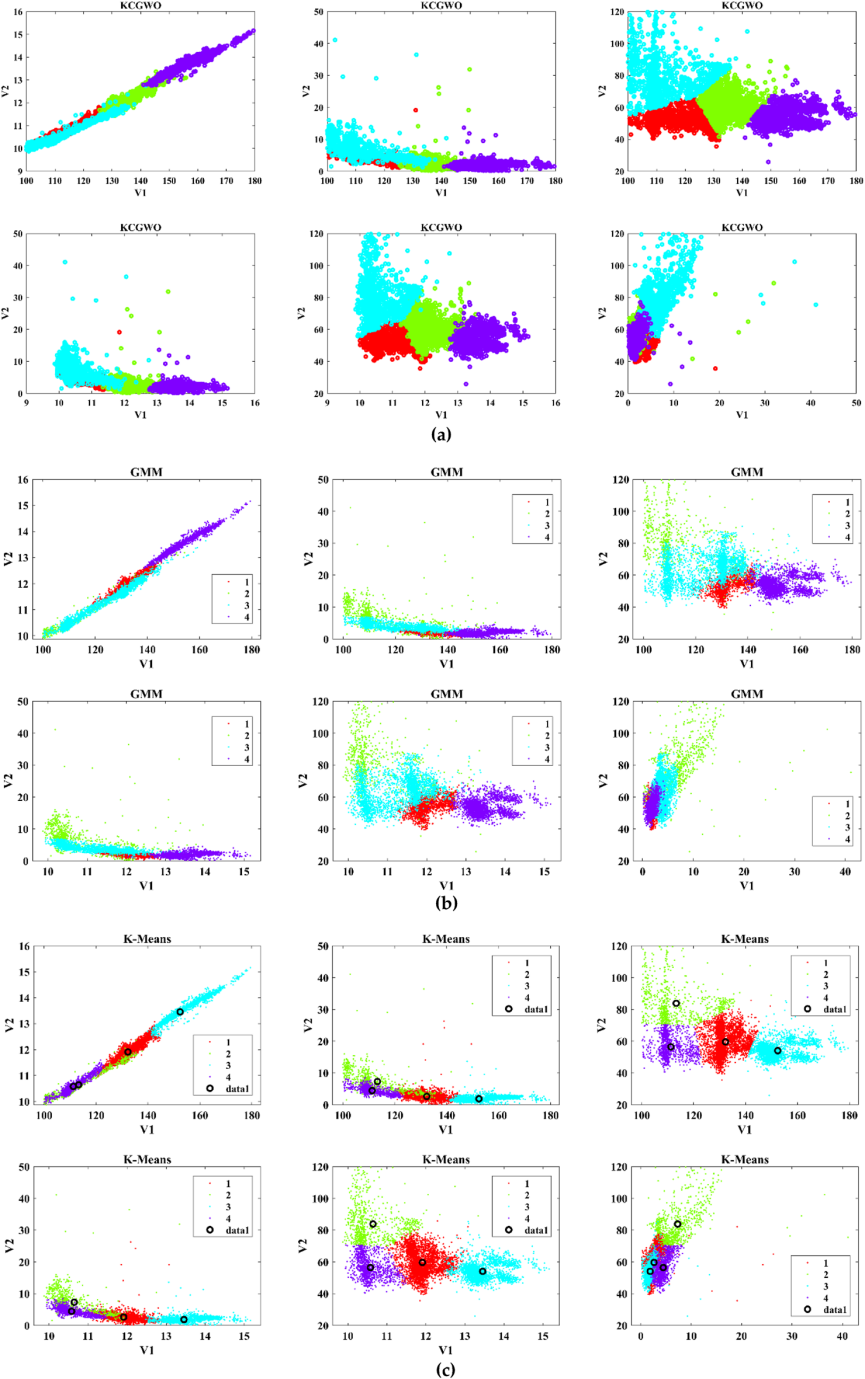
T-SNE plots of emission data; (**a**) KCGWO, (**b**) GMM, (**c**) K-means.

**Figure 6:**
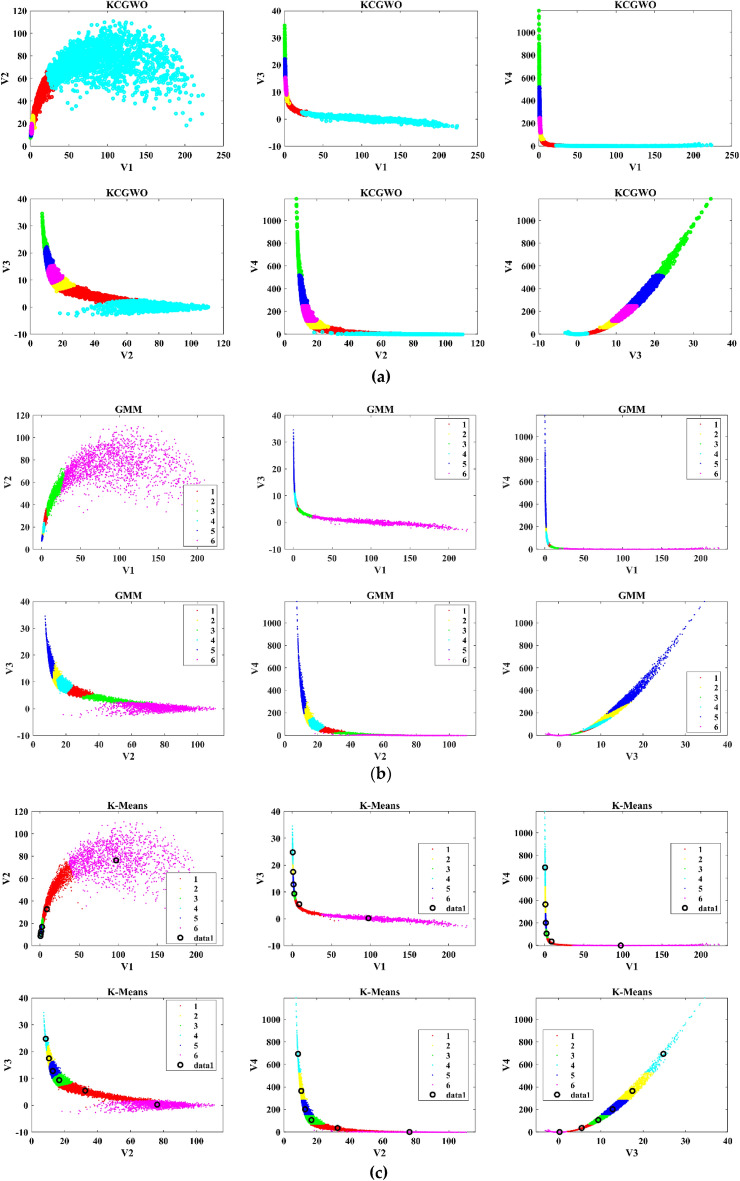
T-SNE plots of HTRU2 data; (**a**) KCGWO, (**b**) GMM, (**c**) K-means.

**Figure 7:**
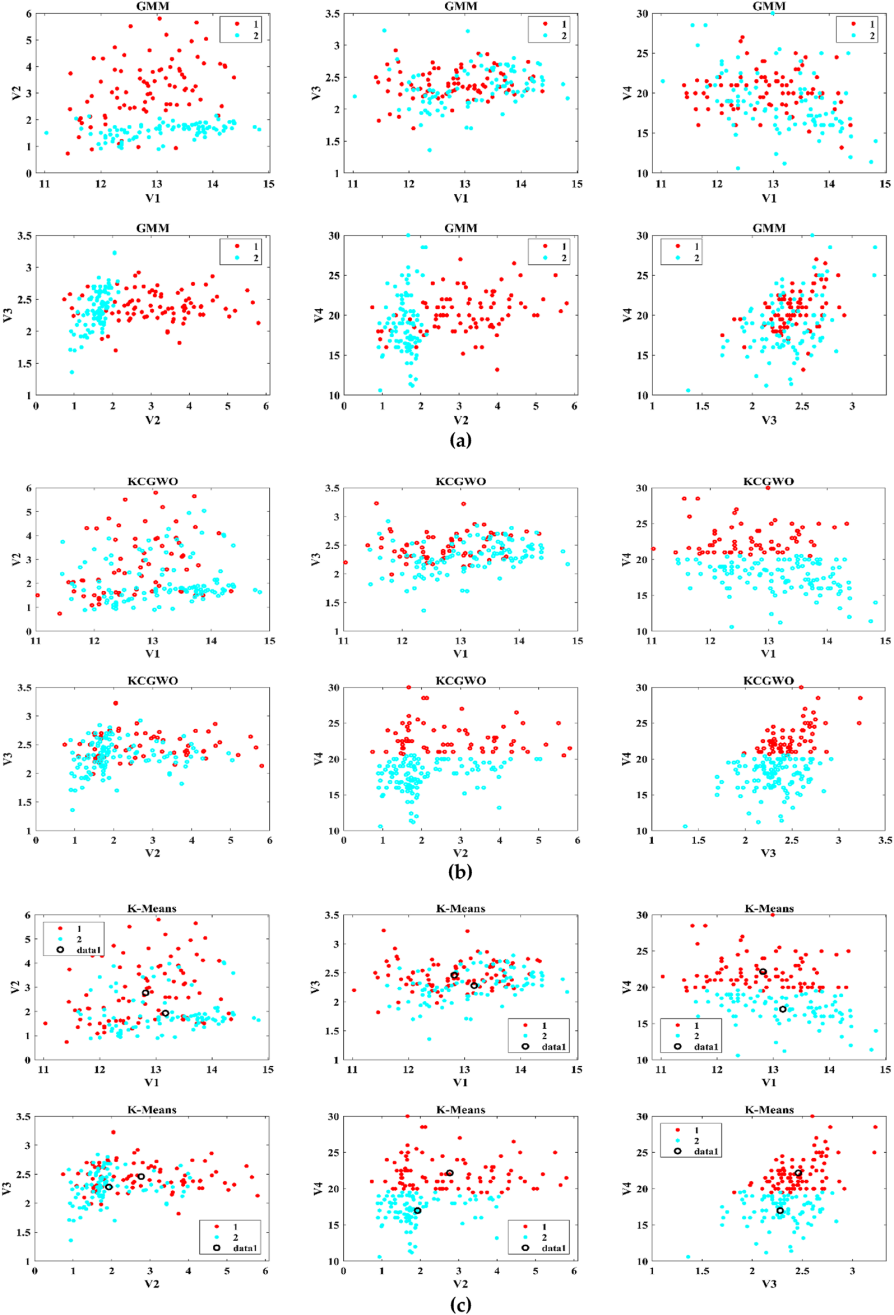
T-SNE plots of Wine data; (**a**) KCGWO, (**b**) GMM, (**c**) K-means.

**Figure 8:**
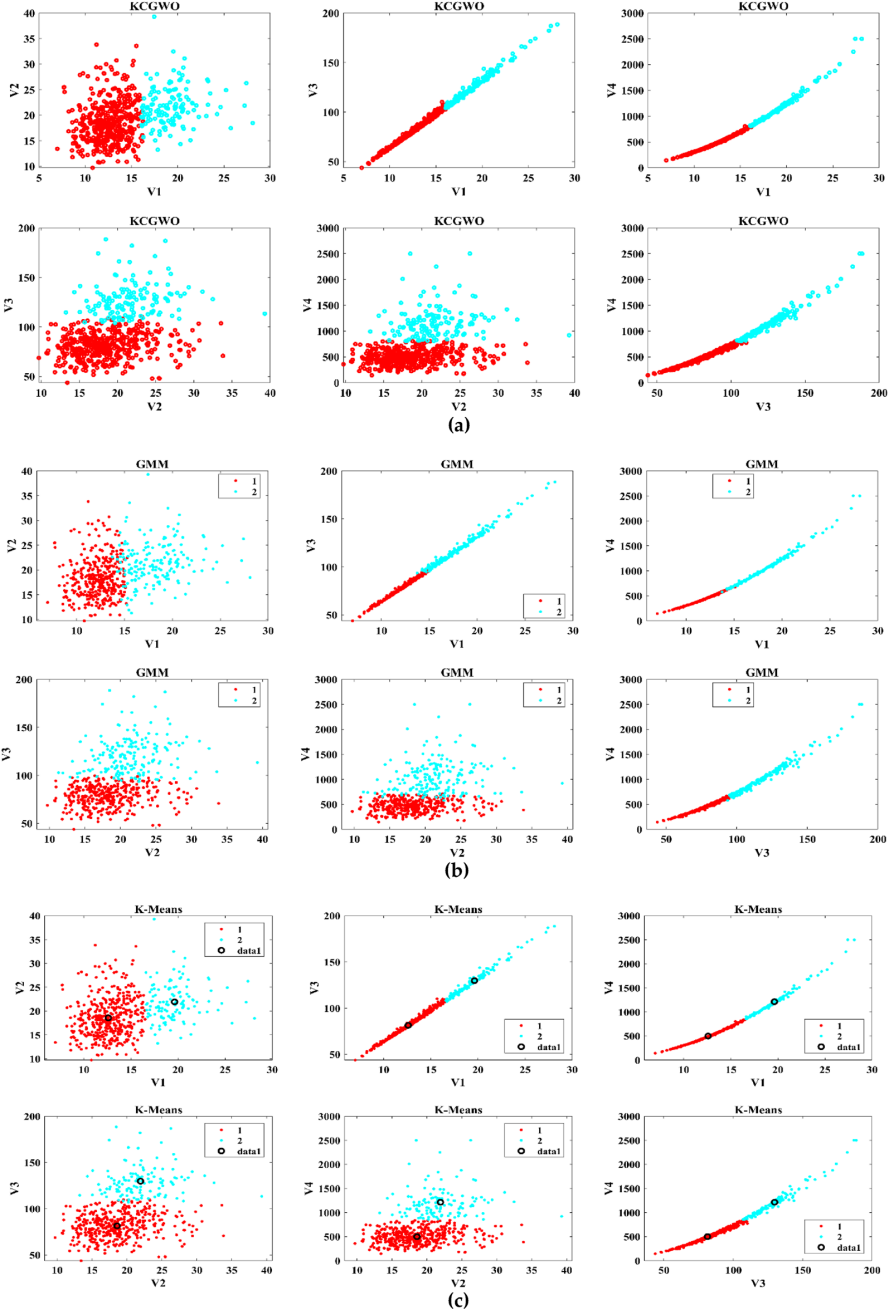
T-SNE plots of Breast cancer data; (**a**) KCGWO, (**b**) GMM, (**c**) K-means.

**Figure 9:**
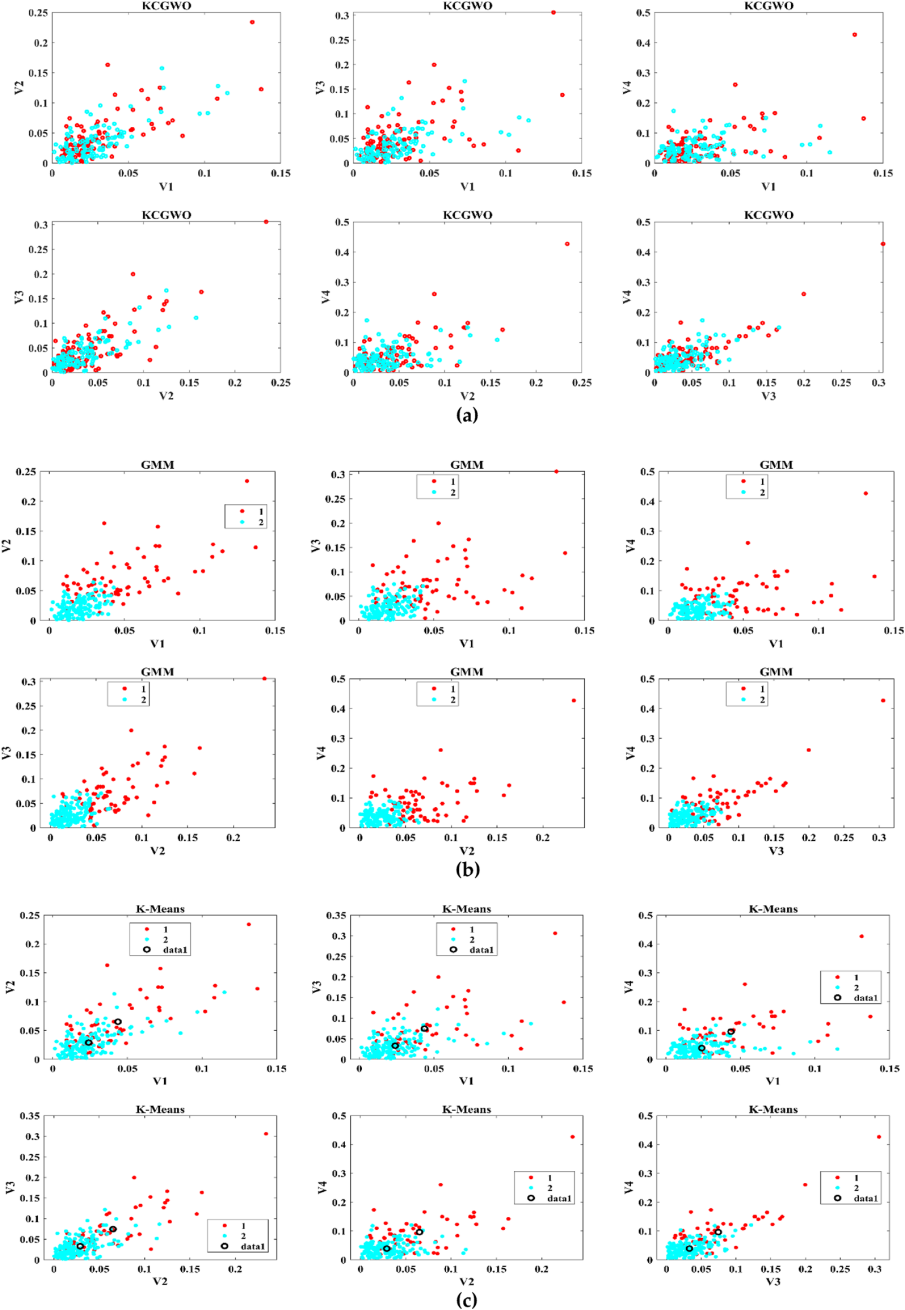
T-SNE plots of Sonar data; (**a**) KCGWO, (**b**) GMM, (**c**) K-means.

**Figure 10:**
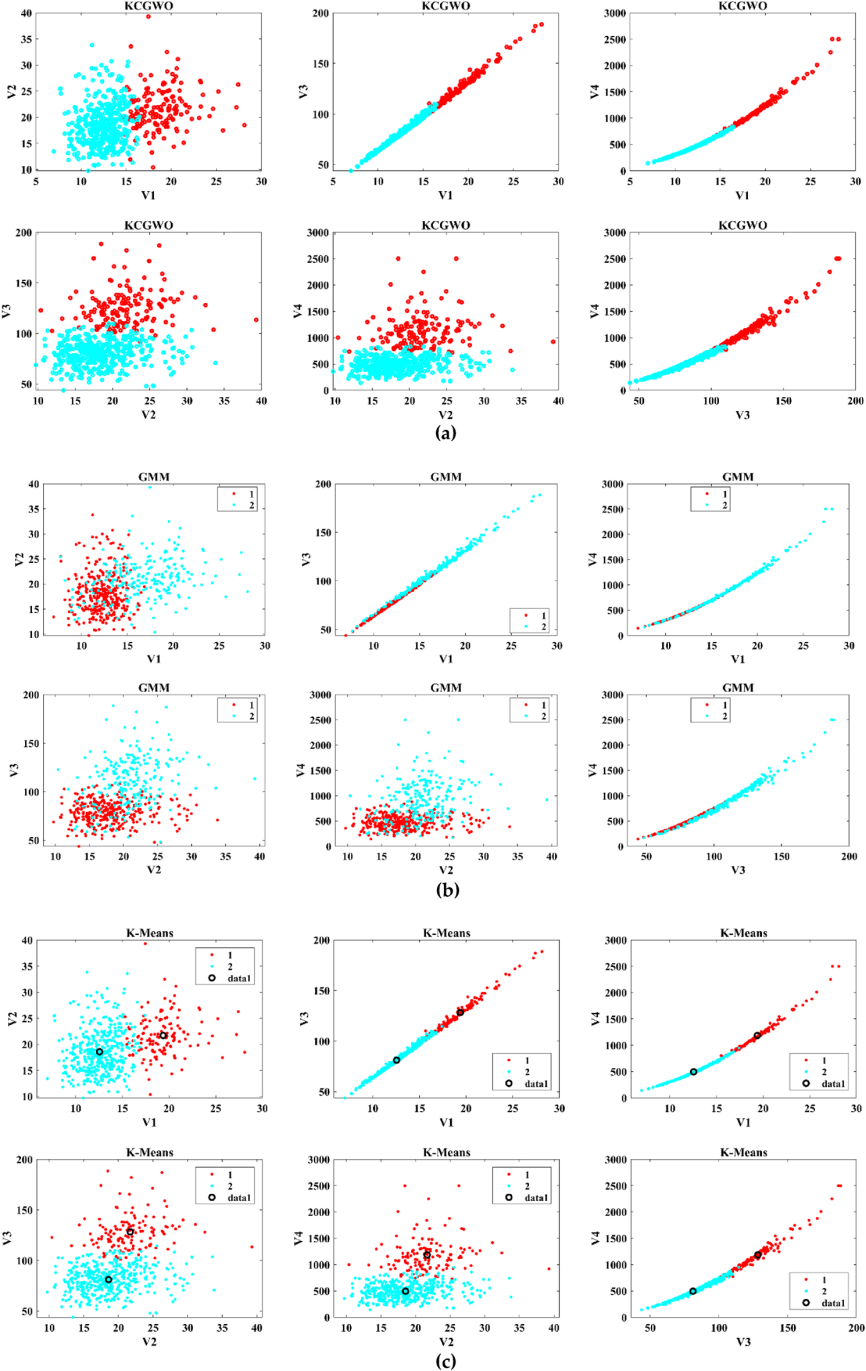
T-SNE plots of WDBC data; (**a**) KCGWO, (**b**) GMM, (**c**) K-means.

**Figure 11:**
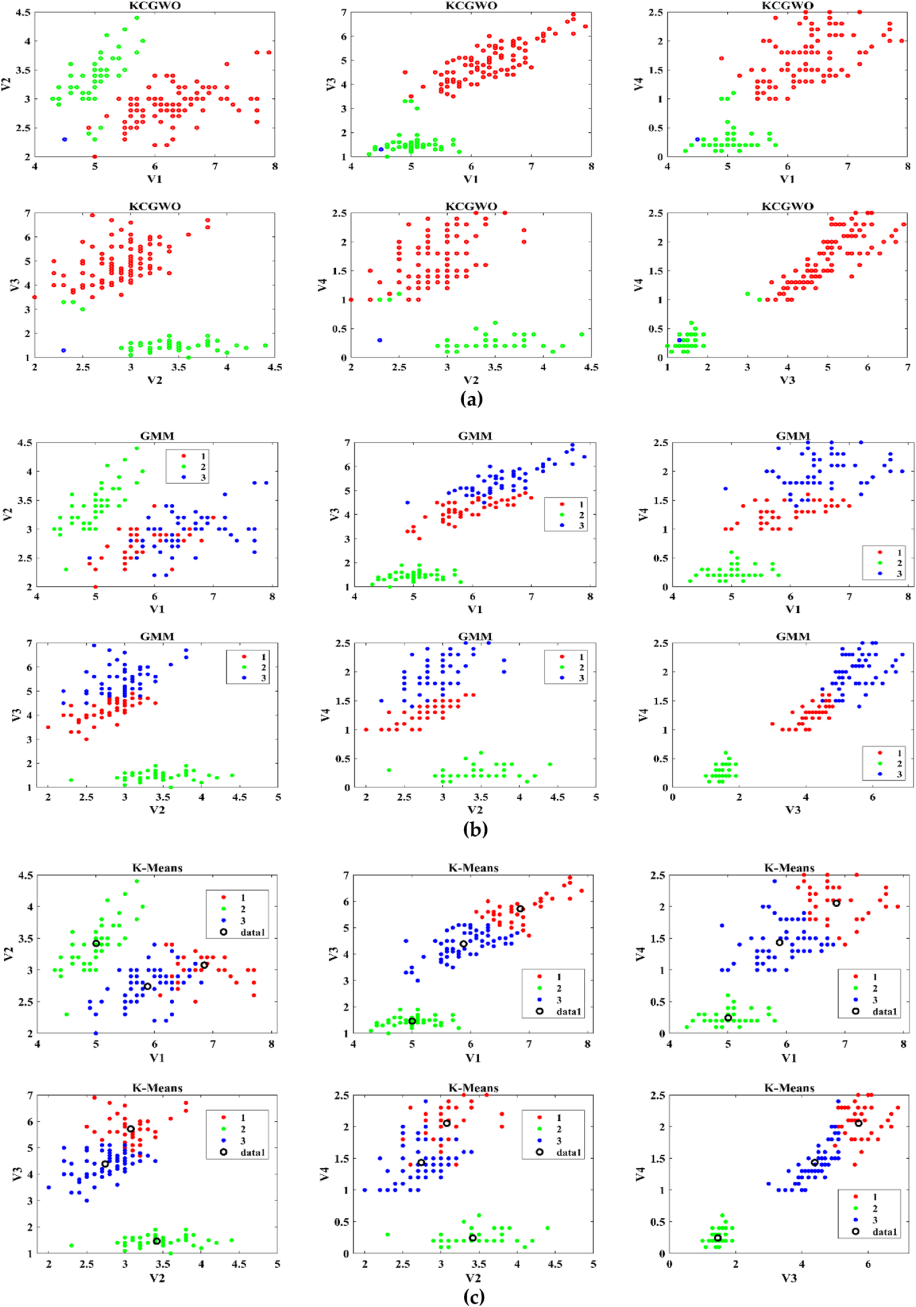
T-SNE plots of iris data; (**a**) KCGWO, (**b**) GMM, (**c**) K-means.

**Figure 12:**
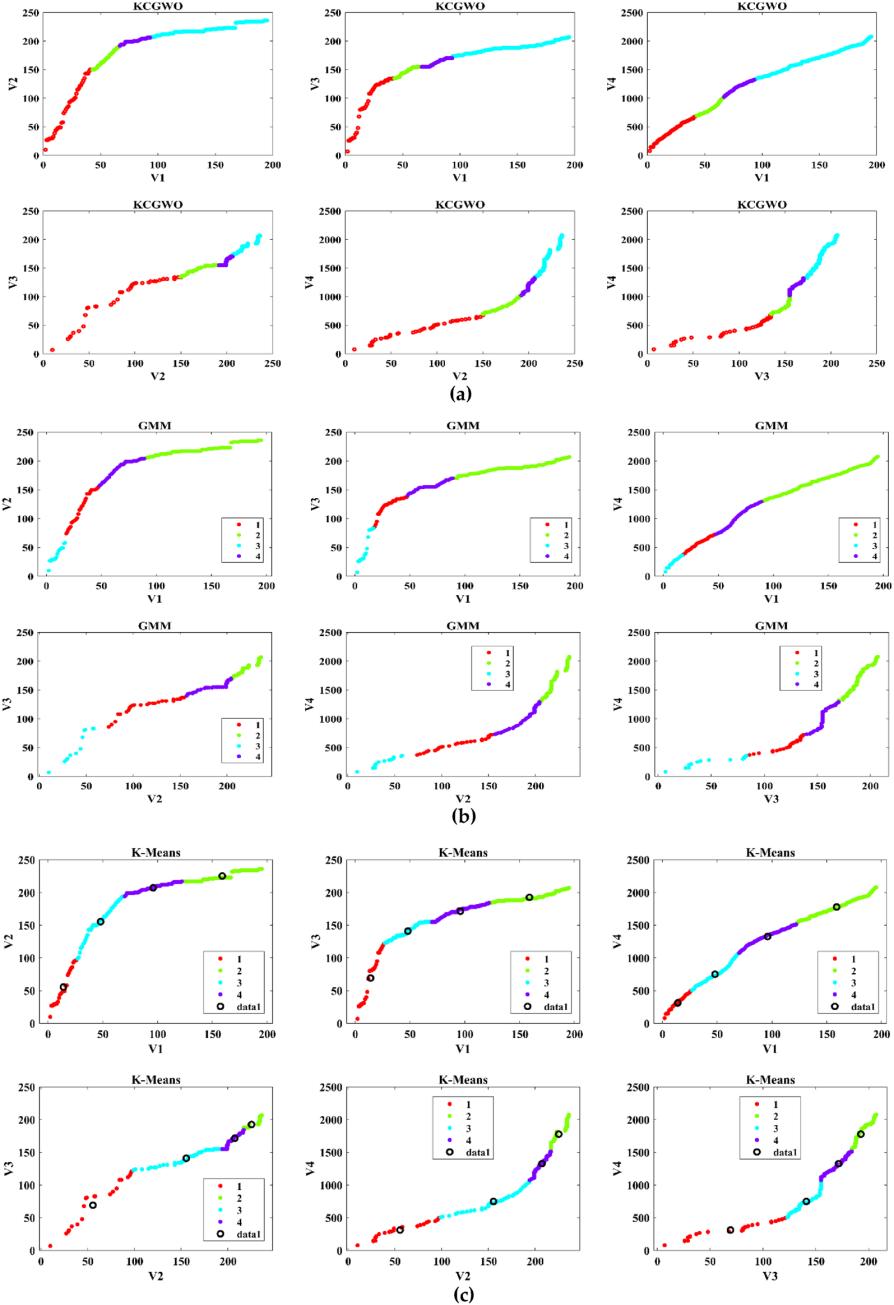
T-SNE plots of 2022 Ukraine-Russia war data; (**a**) KCGWO, (**b**) GMM, (**c**) K-means.

The performance of the proposed KCGWO for clustering is initially compared with standalone K-means clustering algorithms and the Gaussian Mixture Model (GMM). The non-linear, unsupervised t-distributed Stochastic Neighbor Embedding (t-SNE) is typically employed for data analysis and high-dimensional data visualization. For all the selected datasets, t-SNE plots obtained by KCGWO, GMM, and K-means are plotted in Figs. 5, 6, 7, 8, 9, 10, 11 and 12. Figure 5a displays the emission data distribution obtained by the KCGWO between various dimensions. It also shows how well the high-dimensional data are distributed in 2-dimensions. Figure 5b and c show the t-SNE plots obtained by the GMM and K-means algorithms. The cluster centre of each cluster found by the K-means algorithm is also demonstrated in Fig. 5c.

Figure 6a displays the HTRU2 data distribution obtained by the KCGWO between various dimensions. It also shows how well the high-dimensional data are distributed in 2-dimensions. Figure 6b and c show the t-SNE plots obtained by the GMM and K-means algorithms. The cluster center of each cluster found by the K-means algorithm is also demonstrated in Fig. 6c. Figure 7a displays the Wine data distribution obtained by the KCGWO between various dimensions. It also shows how well the high-dimensional data are distributed in 2-dimensions. Figures 7b and c show the t-SNE plots obtained by the GMM and K-means algorithms. The cluster center of each cluster found by the K-means algorithm is also demonstrated in Fig. 7c.

Figure 8a displays the Breast cancer data distribution obtained by the KCGWO between various dimensions. It also shows how well the high-dimensional data are distributed in 2-dimensions. Figure 8b and c show the t-SNE plots obtained by the GMM and K-means algorithms. The cluster center of each cluster found by the K-means algorithm is also demonstrated in Fig. 8c. Figure 9a displays the Sonar data distribution obtained by the KCGWO between various dimensions. It also shows how well the high-dimensional data are distributed in 2-dimensions. Figures 9b and c show the t-SNE plots obtained by the GMM and K-means algorithms. The cluster center of each cluster found by the K-means algorithm is also demonstrated in Fig. 9c.

Figure 10a displays the WDBC data distribution obtained by the KCGWO between various dimensions. It also shows how well the high-dimensional data are distributed in 2-dimensions. Figure 10b and c show the t-SNE plots obtained by the GMM and K-means algorithms. The cluster center of each cluster found by the K-means algorithm is also demonstrated in Fig. 10c. Figure 11a displays the Iris data distribution obtained by the KCGWO between various dimensions. It also shows how well the high-dimensional data are distributed in 2-dimensions. Figure 11b and c show the t-SNE plots obtained by the GMM and K-means algorithms. The cluster center of each cluster found by the K-means algorithm is also demonstrated in Fig. 11c.

Figure 12a displays the 2022 Ukraine-Russia war data distribution obtained by the KCGWO between various dimensions. It also shows how well the high-dimensional data are distributed in 2-dimensions. According to Figs. 5, 6, 7, 8, 9, 10, 11 and 12, in data with convex-shaped clusters, KCGWO has been capable of recognizing clusters and discriminating overlap among clusters quite effectively. This demonstrates how clearly defined the differences between the clusters are. According to Figs. 5, 6, 7, 8, 9, 10, 11 and 12, KCGWO could cluster most of the data points accurately despite the high density and large scatter of sample points in the dataset. This shows that KCGWO is resistant to high data volume and dispersion. Additionally, it has been demonstrated that KCGWO performs effectively when dealing with circular clusters in difficult datasets. KCGWO successfully identifies the majority of the curved regions in these datasets. Due to the utilization of the Euclidean distance measure for clustering, the proposed KCGWO has not completely distinguished all of the clusters in the data.

In order to prove the performance of the proposed KCGWO, two additional metrics, such as Mean Absolute Error (MAE) and Mean Squared Error (MSE), are recorded in Table 7. The average MAE and MSE values obtained by KCGWO with respect to GMM and K-means are listed in Table 7. Based on the average values, it is observed that the performance of the KCGWO with respect to K-means is better than GMM. Based on the comparison of the GMM with respect to K-means, it is observed that GMM is performing better than the K-means clustering algorithm. The results show that KCGWO produced results with more accuracy than GMM and K-means. One way to look at this enhancement is due to the population distribution by K-means and the weight factors, which avoids early convergence and strikes a compromise between global and local searches. Conversely, the proposed KCGWO has significantly enhanced effectiveness in data with significant overlapping and difficulty. As a result, KCGWO outperformed GMM and K-means and improved the ability to identify non-linear clusters.

**Table 7:** MAE and MSE values obtained by KCGWO, GMM, and K-means.

Dataset	MAE				MSE			
	KCGWO vs GMM	KCGWO vs K-means	GMM vs K-means	KCGWO vs K-means (centers)	KCGWO vs GMM	KCGWO vs K-means	GMM vs K-means	KCGWO vs K-means (centers)
Emission	0.6889	1.1532	0.9347	333.5077	0.9378	1.7564	1.4076	12.6256
HTRU2	1.9047	1.1920	1.3160	39,612.27	5.5686	2.5295	3.1712	96.1929
Wine	0.3876	0.1292	0.3483	0.3215	0.3876	0.1292	0.3483	0.2505
Breast cancer	0.0845	0.0194	0.1039	12.2918	0.0845	0.0194	0.1039	760.4808
Sonar	0.4615	0.5769	0.2115	0.0358	0.4615	0.5769	0.2115	0.0027
WDBC	0.8576	0.0334	0.8313	17.8233	0.8576	0.0334	0.8313	3384.228
Iris	0.7600	0.8000	1.0667	0.6654	1.4933	1.5733	2.1333	1.2273
2022 Ukraine Russia war	0.9536	0.8299	0.8351	275.3480	1.3557	0.9948	1.5567	254,083.1
Average values	0.7623	0.5917	0.7059	5031.533	1.3933	0.9516	1.2204	32,292.26

Further, to have a fair comparison, the performance of the proposed algorithm is also compared with other metaheuristic algorithms, such as GWO, MFO, MVO, and SSA, in terms of the statistical measures, such as Min, Mean, Max, and STD. For all algorithms, the population size is carefully chosen as the number of clusters multiplied by 2, and the iteration count is 500. Table 8 recorded all the statistical measures of all selected algorithms and datasets. It is noticed from Table 8 that the KCGWO can able to attain the best Min values for all datasets. The proposed algorithm can converge to the global optima and find the best solution. Except for the WDBC dataset, the proposed algorithm's maximum values are better. The mean and STD values obtained by the proposed algorithm are better than any other algorithms for all selected datasets. It means that the reliability of KCGWO is better than any other selected algorithms for all selected datasets. For each dataset, the ranking is provided based on the Min values, and the average rank values are also logged in Table 8. Based on the mean rank values, KCGWO stands first, followed by SSA, GWO, MFO, and MVO.

**Table 8:** Statistical results obtained for clustering problem by all selected algorithms.

Functions	Metrics	KCGWO	GWO	MFO	MVO	SSA
${\text{Emission}}$	Min	**4.671E + 04**	9.651E + 04	1.314E + 05	1.681E + 05	1.268E + 05
	Max	4.731E + 04	1.038E + 05	1.365E + 05	2.280E + 05	1.465E + 05
	Mean	4.731E + 04	1.038E + 05	1.365E + 05	2.280E + 05	1.465E + 05
	STD	7.841E + 02	1.006E + 04	7.692E + 03	5.345E + 04	1.442E + 04
	Rank	**1**	2	4	5	3
${\text{HTRU}}2$	Min	**4.215E + 05**	1.377E + 06	1.390E + 06	1.600E + 06	1.369E + 06
	Max	4.461E + 05	1.806E + 06	1.400E + 06	1.684E + 06	1.395E + 06
	Mean	4.461E + 05	1.806E + 06	1.400E + 06	1.684E + 06	1.395E + 06
	STD	2.174E + 04	3.448E + 05	1.335E + 04	7.098E + 04	1.742E + 04
	Rank	**1**	3	4	5	2
${\text{Wine}}$	Min	**383.0677**	564.2516	574.3487	582.2759	551.9577
	Max	385.4381	588.2155	597.7833	691.7798	587.9902
	Mean	385.4381	588.2155	597.7833	691.7798	587.9902
	STD	3.817709	24.16611	23.8235	124.3448	32.72063
	Rank	**1**	3	4	5	2
Breast cancer	Min	**7.984E + 04**	8.823E + 04	1.411E + 05	1.507E + 05	1.403E + 05
	Max	8.124E + 04	1.040E + 05	1.412E + 05	1.605E + 05	1.405E + 05
	Mean	8.124E + 04	1.040E + 05	1.412E + 05	1.605E + 05	1.405E + 05
	STD	1.753E + 03	1.637E + 04	1.440E + 02	1.392E + 04	2.119E + 02
	Rank	**1**	2	4	5	3
${\text{Sonar}}$	Min	**15.77131**	21.42128	20.34382	19.43976	19.00758
	Max	17.16814	24.3906	20.88503	22.09095	19.71072
	Mean	17.16814	24.3906	20.88503	22.09095	19.71072
	STD	1.60654	2.033542	0.431548	3.517527	0.417388
	Rank	**1**	5	4	3	2
${\text{WDBC}}$	Min	**1.563E + 05**	2.133E + 05	2.684E + 05	2.805E + 05	2.647E + 05
	Max	2.348E + 05	2.741E + 05	2.726E + 05	3.037E + 05	2.681E + 05
	Mean	2.348E + 05	2.741E + 05	2.726E + 05	3.037E + 05	2.681E + 05
	STD	1.129E + 05	5.706E + 04	3.622E + 03	2.202E + 04	3.404E + 03
	Rank	**1**	2	4	5	3
${\text{Iris}}$	Min	**96.68885**	192.0871	292.3649	294.3163	284.2368
	Max	99.58939	248.1072	325.407	335.4001	285.4755
	Mean	99.58939	248.1072	325.407	335.4001	285.4755
	STD	4.793102	39.83272	32.09701	38.01879	0.960675
	Rank	**1**	2	4	5	3
Ukraine Russia war	Min	**5.565E + 04**	2.284E + 05	2.188E + 05	2.317E + 05	2.146E + 05
	Max	5.776E + 04	2.915E + 05	2.582E + 05	2.812E + 05	2.152E + 05
	Mean	5.776E + 04	2.915E + 05	2.582E + 05	2.812E + 05	2.152E + 05
	STD	1.810E + 03	5.386E + 04	2.336E + 04	5.189E + 04	8.246E + 02
	Rank	**1**	4	**3**	5	2
Average rank		**1.000**	2.875	3.875	4.750	2.500

Significant values are in bold.

**Figure 13:**
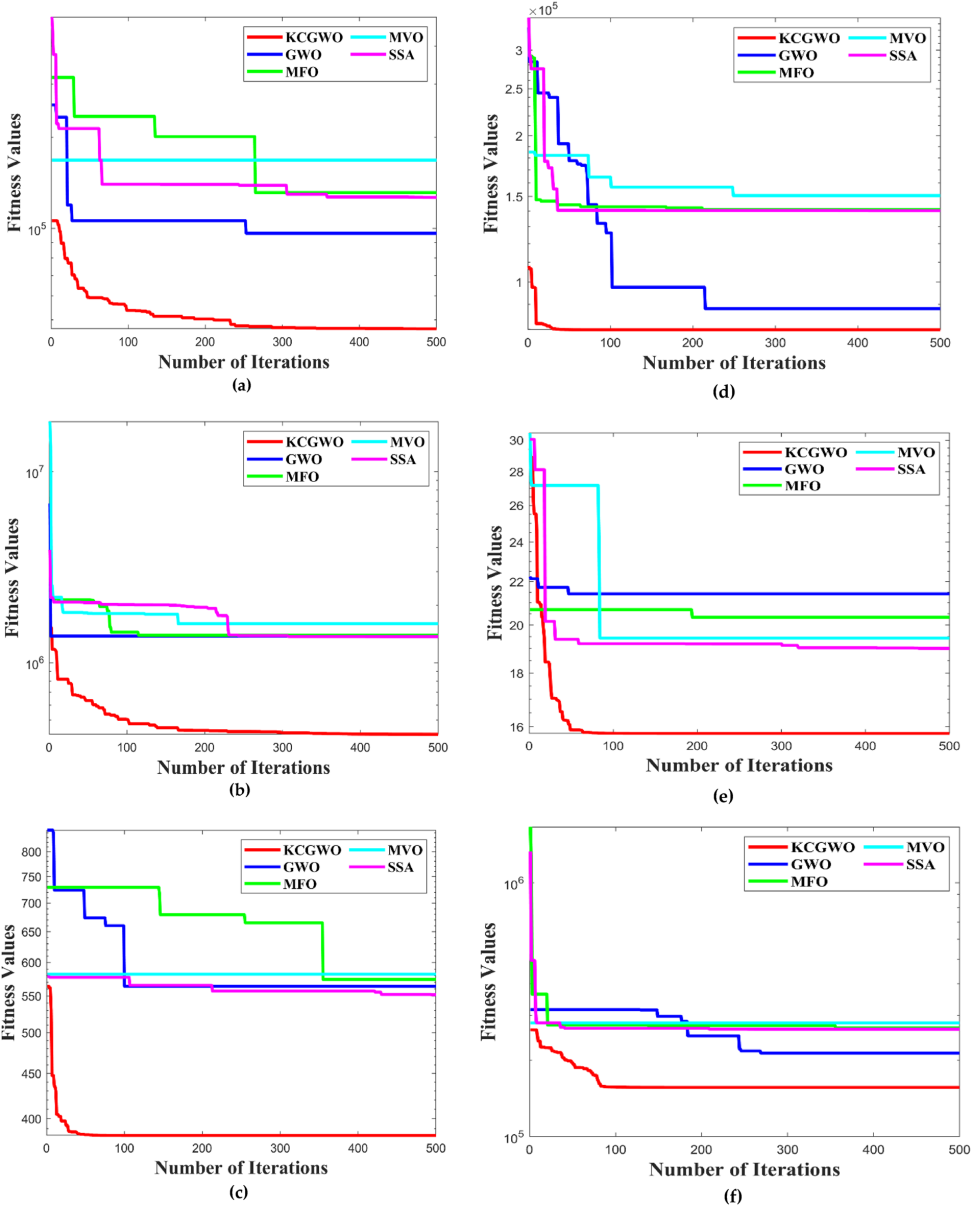

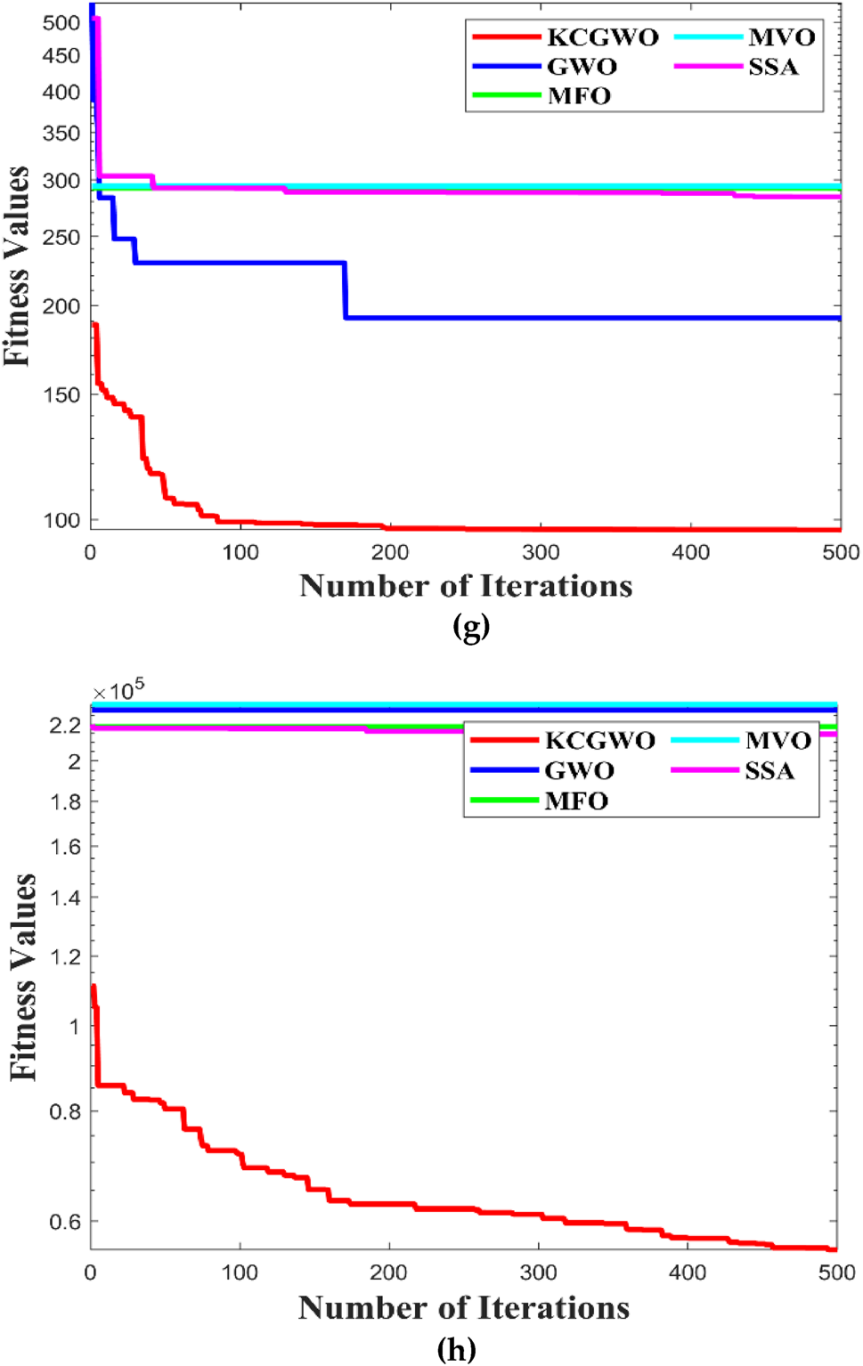
Convergence curves obtained by all algorithms: (**a**) Emission, (**b**) HTRU2, (**c**) Wine, (**d**) Breast cancer, (**e**) Sonar, (**f**) WDBC, (**g**) Iris, (**h**) 2022 Ukraine-Russia war.

**Figure 14:**
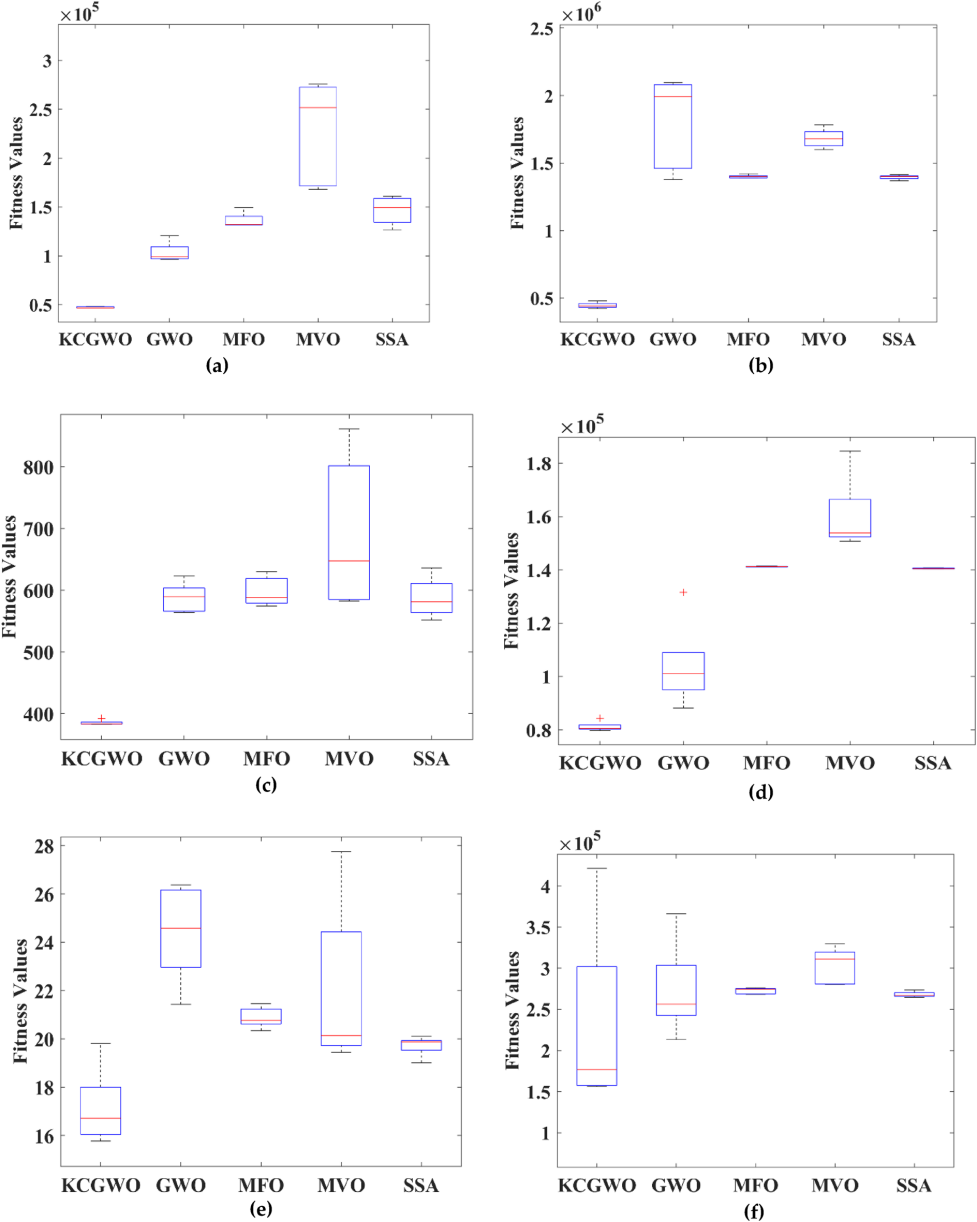

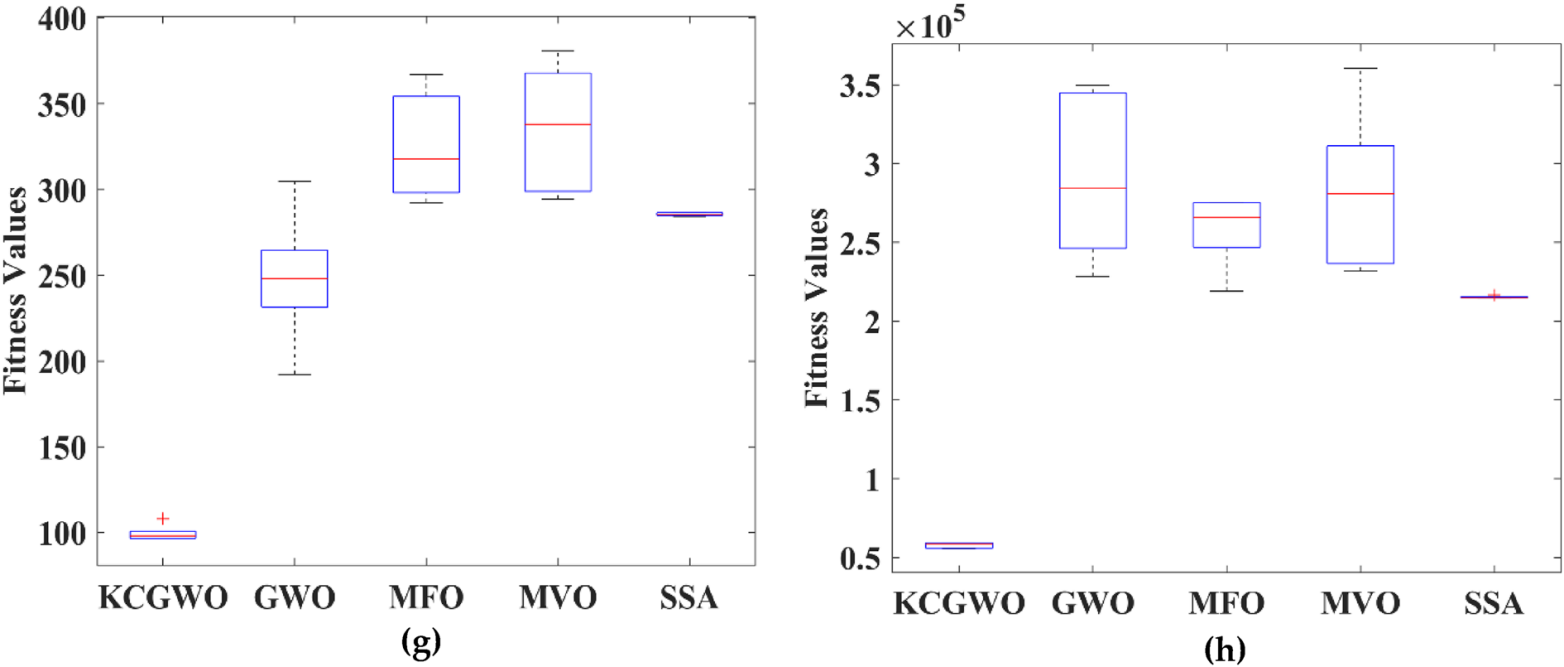
Boxplots obtained by all algorithms; (**a**) Emission, (**b**) HTRU2, (**c**) Wine, (**d**) Breast cancer, (**e**) Sonar, (**f**) WDBC, (**g**) Iris, (**h**) 2022 Ukraine-Russia war.

The following ratios of sequential errors describe the convergence rate given an undetermined optimal value, which is typically the case in data clustering applications:

18 $$\mathrm{Convergence \,\,Rate} \left(CR\right)=\frac{\left|{f}_{i+1}-{f}_{i}\right|}{\left|{f}_{i}-{f}_{i-1}\right|} ,$$

where ${f}_{i}$ denotes the fitness value during the current iteration, ${f}_{i+1}$ denotes the fitness value during the next iteration, and ${f}_{i-1}$ denotes the fitness value during the previous iteration. The logarithmic CR plot measures the dynamic fitness change all over the iteration. The curves of logarithmic convergence curves are illustrated in Fig. 13 to visualize the effect on the various datasets. Comparatively to the other configurations, such as GWO, MFO, MVO, and SSA, using K-means clusters with weight factors in GWO has produced a good convergence that avoids the local optimum trap, with the lowest MAE and MSE values occurring at iteration 500. The adopted mechanism in the GWO algorithm maintained a reasonable balance between them and produced suitable population patterns for exploration and exploitation. In addition to the convergence curve, the boxplot analysis is also made to prove the reliability of the algorithms selected. All the algorithms are executed 30 times. The boxplots are plotted and illustrated based on the recorded values in Fig. 14.

From Fig. 14, it is clearly evident that the reliability of the KCGWO is superior to all the selected algorithms. The computational time necessary by the algorithm to find the overall optimal solution is known as the time to best solution. The RT of an algorithm is the sum of all computations performed until its stopping criterion stops it. Therefore, the RT is recorded for the selected algorithms and recorded in Table 9.

**Table 9:** RT values of all algorithms for the clustering problem.

Functions	KCGWO	GWO	MFO	MVO	SSA
${\text{Emission}}$	2.145	2.047	2.569	2.602	2.554
${\text{HTRU}}2$	3.419	3.217	3.987	4.001	3.984
${\text{Wine}}$	1.265	1.193	1.547	1.698	1.654
Breast cancer	2.413	2.341	2.974	3.047	2.998
${\text{Sonar}}$	2.987	2.801	3.014	3.142	3.131
WDBC	2.545	2.471	2.698	2.774	2.709
Iris	1.016	0.998	1.262	1.411	1.345
Ukraine Russia war	1.169	1.045	1.335	1.406	1.347
Average RT	2.120	**2.014**	2.423	2.510	2.465

Significant values are in bold.

Similar to numerical optimization problems, the average RT values are provided in Table 9, and based on the mean RT value, the original GWO has less RT value, and the RT value of KCGWO is slightly greater than the GWO, which is due to the fact that the introduction of the K-means clustering mechanism. At the same time, the weight factor does not impact the proposed algorithm's computational complexity. It is clear from the prior comparisons and discussions that the improvisation of GWO performance with K-Means clustering and weight factors has accomplished its objectives and improved the original GWO algorithm. The new adjustments enabled KCGWO to defeat numerous original and other selected algorithms, presenting KCGWO as a global optimizer and an efficient data clustering technique that can be applied in industrial applications.

### Discussions

While the KCGWO introduces significant improvements to the conventional GWO, enhancing its applicability to data clustering tasks, it is not without its limitations. These constraints, inherent to the methodology and application context, warrant consideration for future research and practical implementation. KCGWO's performance is partly contingent on the initial clustering obtained from the K-means algorithm. This dependence means that the quality of KCGWO's outcomes can be affected by the initial positioning of centroids in K-means, which is sensitive to the chosen initial points. If the K-means algorithm converges to a local optimum during its initialization phase, KCGWO may start from a less advantageous position, potentially impacting the overall optimization process. The introduction of a dynamic weight factor in KCGWO, while beneficial for balancing exploration and exploitation, adds complexity in terms of parameter tuning. The performance of KCGWO can be sensitive to the settings of this weight factor alongside other algorithm parameters. Finding the optimal configuration requires extensive experimentation and can be computationally demanding, especially for large-scale problems or datasets with high dimensionality. Although KCGWO is designed to explore and exploit the solution space efficiently, the computational overhead introduced by the integration of K-means and the dynamic weight adjustment mechanism can increase the algorithm's computational complexity. This may limit the scalability of KCGWO to very large datasets or real-time clustering applications where computational resources or time are constrained. While empirical tests have demonstrated KCGWO's effectiveness on various datasets, its ability to generalize across all types of data distributions remains a concern. The algorithm's performance on datasets with complex structures, high dimensionality, or noise could vary, and its robustness in these scenarios has not been fully explored. The K-means component of KCGWO may not be inherently robust against noise and outliers, as K-means tends to be influenced by these factors. Consequently, KCGWO's performance could be degraded in datasets where noise and outliers are prevalent, affecting the quality of the clustering outcomes.

Addressing these limitations presents paths for future work, including the development of strategies to reduce dependence on initial clustering quality, adaptive parameter tuning mechanisms to mitigate sensitivity issues, and enhancements to computational efficiency. Additionally, further research could explore the incorporation of noise and outlier handling techniques to improve the robustness of KCGWO across diverse and challenging data environments.

## Conclusions

This study advances data clustering and optimization through the development of an innovative approach, integrating the GWO with K-Means clustering, further augmented by a dynamic weight factor mechanism. This integration not only contributes to the theoretical framework of swarm intelligence methods but also demonstrates practical applicability in enhancing data clustering outcomes. The theoretical implications of this research are underscored by the systematic incorporation of a traditional clustering algorithm with a contemporary optimization technique, enriching the metaheuristic algorithm landscape. This methodology offers a new perspective on achieving a balance between exploration and exploitation in swarm-based algorithms, a pivotal factor in their efficiency and effectiveness for complex problem-solving. From a practical perspective, the introduction of the KCGWO represents a significant advancement towards more accurate and efficient data clustering solutions. By ingeniously adjusting swarm movements based on initial positions and integrating weight factors, the method exhibits enhanced diversity and an improved ability to escape local optima. These features are essential for applications demanding precise data segmentation, such as image recognition, market segmentation, and biological data analysis.

The contributions of this research extend beyond theoretical enhancement, offering tangible benefits to sectors reliant on data analytics. The improved exploration and exploitation dynamics of KCGWO result in faster convergence rates and superior clustering outcomes, rendering it an invaluable asset for processing large datasets with intricate structures. This is particularly pertinent in the Big Data context, where rapid and accurate clustering of large data sets can significantly influence decision-making processes and resource management.

In summary, the KCGWO algorithm marks a notable academic contribution to the discourse on optimization algorithms and facilitates its application across various practical scenarios. Its adaptability and efficiency herald new possibilities for addressing data-clustering challenges in diverse fields, signalling a new era of optimization solutions that are robust and responsive to the dynamic requirements of data analysis.

## Data Availability

The dataset used in this paper is available in open source at https://archive.ics.uci.edu/datasets?Task=Clustering. All other data is included in the paper, and no additional data has been used in this study.
