# Author Correction: Augmented weighted K-means grey wolf optimizer: An enhanced metaheuristic algorithm for data clustering problems

**DOI:** 10.1038/s41598-024-58099-3

**Published:** 2024-03-27

**Authors:** Manoharan Premkumar, Garima Sinha, Manjula Devi Ramasamy, Santhoshini Sahu, Chithirala Bala Subramanyam, Ravichandran Sowmya, Laith Abualigah, Bizuwork Derebew

**Affiliations:** 1 Department of Electrical & Electronics Engineering, Dayananda Sagar College of Engineering, Kumaraswamy Layout, Bengaluru, Karnataka 560078 India; 2 Department of Computer Science and Engineering, Jain University, Ramanagaram, Bengaluru, Karnataka India; 3 Department of Computer Science and Engineering, KPR Institute of Engineering and Technology, Coimbatore, Tamil Nadu India; 4 Department of Computer Science & Engineering, GMR Institute of Technology, Rajam, Srikakulam, Andhra Pradesh India; 5 Department of Computer Science and Engineering, Vardhaman College of Engineering, Hyderabad, India; 6 Department of Electrical and Electronics Engineering, Manipal Institute of Technology, Manipal Academy of Higher Education, Manipal, Karnataka India; 7 Computer Science Department, Al al-Bayt University, Mafraq, 25113 Jordan; 8 Artificial Intelligence and Sensing Technologies (AIST) Research Center, University of Tabuk, 71491 Tabuk, Saudi Arabia; 9 Hourani Center for Applied Scientific Research, Al-Ahliyya Amman University, Amman, 19328 Jordan; 10 MEU Research Unit, Middle East University, Amman, 11831 Jordan; 11 Department of Electrical and Computer Engineering, Lebanese American University, Byblos, 13-5053 Lebanon; 12 School of Engineering and Technology, Sunway University Malaysia, 27500 Petaling Jaya, Malaysia; 13 College of Engineering, Yuan Ze University, Taoyuan, Taiwan; 14 Applied science research center, Applied science private university, Amman, 11931, Jordan; 15 Department of Statistics, College of Natural and Computational Science, Mizan-Tepi University, Tepi Bushira, Ethiopia

Correction to: *Scientific Reports* 10.1038/s41598-024-55619-z, published online 05 March 2024

In the original version of this Article, Laith Abualigah and Bizuwork Derebew were incorrectly affiliated. The correct affiliation is listed below.

Laith Abualigah

- ‘Computer Science Department, Al al-Bayt University, Mafraq 25113, Jordan.’
- ‘Artificial Intelligence and Sensing Technologies (AIST) Research Center, University of Tabuk, 71491 Tabuk, Saudi Arabia.’
- ‘Hourani Center for Applied Scientific Research, Al-Ahliyya Amman University, Amman 19328, Jordan.’
- ‘MEU Research Unit, Middle East University, Amman 11831, Jordan.’
- ‘Department of Electrical and Computer Engineering, Lebanese American University, Byblos 13-5053, Lebanon.’
- ‘School of Engineering and Technology, Sunway University Malaysia, 27500 Petaling Jaya, Malaysia.’
- ‘College of Engineering, Yuan Ze University, Taoyuan, Taiwan.’
- ‘Applied science research center, Applied science private university, Amman, 11931, Jordan.’

Bizuwork Derebew

- ‘Department of Statistics, College of Natural and Computational Science, Mizan-Tepi University, Tepi Bushira, Ethiopia.’

The original Article has been corrected.

